# Supplementary material for: Associations between common genetic variants and income provide insights about the socio-economic health gradient
Source: Nat Hum Behav. 2025 Jan 28;9(4):794–805. doi: 10.1038/s41562-024-02080-7 (PMC12018258; doi:10.1038/s41562-024-02080-7)
Supplement: Supplementary file 1 — Supplementary Information, the FAQ document and Supplementary Figs. 1–3. [file 41562_2024_2080_MOESM1_ESM.pdf]

nature human behaviour

Supplementary information

https://doi.org/10.1038/s41562-024-02080-7

Associations between common genetic variants and income provide insights about the socio-economic health gradient

In the format provided by the authors and unedited

Table of contents

1. Study Overview 3
2. GWAS, quality control, and meta-analysis 3
2.1. Phenotype definition and construction 4
2.1.1. General definition 4
2.1.2. Individual income 5
2.1.3. Household income 5
2.1.4. Occupational income 5
2.1.4.1. UK Biobank and ALSPAC mothers 5
2.1.4.2. Lifelines and Netherlands Twin Registry 6
2.1.4.3. Estonian Genome Center 6
2.1.4.4. HUNT 7
2.1.5. Parental income (iPSYCH) 7
2.2. Genotyping and imputation 7
2.3. Association analyses 7
2.4. Quality control 8
2.5. Meta-analysis 8
2.5.1. Step 1: Sex-Stratified Meta-Analysis Using METAL 8
2.5.2. Step 2: Combining Male and Female Results with MTAG 9
2.5.3. Step 3: Combining Different Income Measures with MTAG 9
2.5.4. Step 4: Final SNP Selection and Effective Sample Size Calculation 9
2.5.5. Evaluation of Meta-Analysis Results 9
2.5.6. Effective Sample Size Estimation 10
2.5.7. Bias Correction and Effect Size Measurement 10
2.6. Identification of genomic loci 10
2.7. Winner's-curse adjustment 11
3. Environmental heterogeneity 11
3.1. Between-sex heterogeneity 11
3.2. Cross-country heterogeneity 11
4. Comparison with educational attainment 12
4.1. Biological annotation 12
4.2. LDSC and MiXeR 13
4.3. GWAS-by-subtraction 13
4.4. Concordant and discordant sets 14
5. Polygenic score analyses of income 15
5.1. Baseline polygenic prediction 15
5.1.1. The STR income data description 16
5.2. Within-family polygenic prediction 17
5.3 Polygenic prediction in samples of non-European descent 18
6. Genetic correlation analysis 19
7. Biological annotation 19
7.1. Gene mapping 19
7.2. Tissue-specific enrichment analysis 19
8. GREML heritability estimation 20
9. Phenome-wide association study 20
10. Cohort acknowledgements 20
10.1. ALSPAC (Avon Longitudinal Study of Parents and Children) 20
10.2. CoLaus (Cohorte Lausannoise) 21
10.3. Croatia - Korcula 22
10.4. EGCUT (Estonian Genome Center, University of Tartu) 22
10.5. FTC (Finnish Twin Cohort) 22

10.6. HUNT (Trøndelag Health Study) 22
10.7. iPSYCH 23
10.8. LifeLines 23
10.9. MOBA (Norwegian Mother, Father and Child Cohort Study) 23
10.10. NEO (The Netherlands Epidemiology of Obesity Study) 24
10.11. NTR (Netherlands Twin Registry) 24
10.12. QIMR (Queensland Institute of Medical Research) 24
10.13. RS (Rotterdam Study) 24
10.14. SHIP (Study of Health in Pomerania) 25
10.15. STR (Swedish Twin Registry) 25
10.16. UKHLS (Understanding Society) 25
11. References 26
1. Frequently Asked Questions (FAQs) 29
2. Summary 30
3. The current study 32
3.1. What is the purpose of this study? 32
3.2. What did you do in this paper? 33
3.3. How was income measured in the current study? 33
3.4. Did you find the gene(s) for income? 34
3.5. Are the genetic variants associated with income in your study also associated with other outcomes? 35
3.6. How good is your polygenic index? 35
3.7. What does your study *not* mean? 36
3.8. Is this the first GWAS on income? 37
4. Implications of the study 38
4.1. Does this study show that an individual's level of income is determined, or fixed, at conception? 38
4.2. Can your polygenic index be used to predict how well someone will do in life? 38
4.3. Can your polygenic index be used for research studies in non-European-ancestry populations? 40
4.4. What policy lessons do you draw from this study? How could society benefit from this work? 41
4.5. Could this kind of research lead to discrimination against, or stigmatization of, people with the relevant genetic variants? If so, why conduct this research? 42
5. Background 44
5.1. What does it mean to say that income is "heritable"? 44
5.2. What is a GWAS? Are the genetic variants identified in a GWAS "causal"? 44
5.3. What is a polygenic index? 47
5.4. Where can I learn more about social science genetics? 48
6. Appendices 49
6.1. Quality control measures 49
6.2. Additional reading and references 50
Supplementary Fig. 1. Effect sizes of Income Factor GWAS 54
Supplementary Fig 2. Polygenic prediction of income 55
Supplementary Fig. 3. Polygenic prediction of income in samples of non-European ancestries 56

1. Study Overview

We conducted a genome-wide association study (GWAS) of income, using four income measures and data collected from more than 600,000 participants in 26 cohorts from 12 countries. Due to data availability and statistical power considerations, our analyses were restricted to individuals carrying genotypes most similar to the EUR panel of the 1000 Genomes data set (1KG-EUR), as compared to individuals samples elsewhere in the world. The meta analysis across cohort-level results was carried out per each income measure and then the results across the four income measures were combined by extracting their shared genetic basis. Then a number of follow-up analyses were conducted. First, we investigated the environmental heterogeneity between sex and between countries. Second, we examined the shared genetic factor with educational attainment (EA). Third, we performed polygenic prediction analyses. Fourth, we estimated the genetic correlation of income with a number of other related phenotypes and compared the results with the estimates for EA. Finally, we performed brief biological annotation analyses.

This study was carried out under the auspices of the Social Science Genetic Association Consortium (https://www.thessgac.org/).

2. GWAS, quality control, and meta-analysis

We pre-registered our analysis plan for the main income GWAS meta-analysis on August 30 2018 (https://osf.io/rg8sh/). In total, we recruited 31 cohorts, which have one of the following income measures available: individual, occupational household, and parental income. Some of these cohorts contributed to multiple income measures.

The sample inclusion criteria according to our analysis plan are as follows:

1. Samples are of European ancestry (1KG-EUR-like individuals);
2. They are finished with education. If such information is unavailable, limit analyses to those aged >30 years
3. All relevant covariates are available for the individual
4. They were successfully genotyped genome-wide (recommended individual genotyping rate: > 95%).
5. They passed the cohort-specific standard quality controls (e.g., excluding individuals who are genetic outliers in the cohort).
6. In the case of self-reported income, unreasonable answers should be removed (e.g., negative income or yearly income > 10 mio EUR). The number of deleted observations and the respective reason for deletion as well as income histograms need to be reported in the descriptive statistics summary file of the cohort.

2.1. Phenotype definition and construction

Individual income is the result of various factors including achieved qualifications (e.g. education, learnt occupation, experience), personal characteristics (e.g. leadership, cognitive skills, consciousness), the demand and supply for these qualifications and characteristics in the labor market, and personal choices about labor supply (e.g. due to personal preferences, decisions about division of labor among household members). In this paper, we aimed to study the genetic factor for such individual earning potential. For this purpose, it was ideal to use individual income measures. However, individual income information was typically not collected in most of the genotyped samples. To circumvent such empirical challenges, we used four measures of income (individual, occupational, household, and parental income) and conducted a multivariate GWAS to combine these different measures. **Supplementary Tables 1-2** summarize the details of income measures used for each cohort.

2.1.1. General definition

For all income measures considered, we defined the main phenotype as the natural log of income before-tax. It is important to use the log transformation here because this allows us to correct for the typical skewness of the income distribution, which will return a better linear fit, as well as to model the percentage change in income, which is unit-free. Ideally, the phenotype included all "earned" financial compensation (salaries, income from self-employment, profits from running one's own business, bonuses, vacation benefits) but excluded non-earned monetary transfers such as rental income, capital gains, dividends, and transfers from the government, family, or former spouses.

Many cohorts opted to use categorical responses to measure individual or household income. In these cases, we converted these categories to a semi-continuous measure by taking the natural logarithm of the midpoint of the category. As the top and bottom category are often open-ended and do not have a midpoint, we converted the top category by taking the logarithm of 4/3 times the lower bound of that category and the bottom category by taking the logarithm of 3/4 times the upper bound of that category.

When multiple observations of the income measure per individual were available (i.e. longitudinal data), we first regressed the income measure on all control variables including time-specific intercepts. Then, the mean of the residuals for each person were taken as the phenotype.

Some of the cohorts of older adults had a large share of retired individuals who may have been receiving pension. For these individuals, we used their last observed wage. If their last wage was not available, we derived occupational wage from their last occupation. In either case, they were treated as if they were observed while they had their last job. For instance, if a 65-year-old retired individual was surveyed in 2009 and her past wage or occupational wage for the job that she had in 2006 was available, her age and year of observation was 62 and 2006, respectively, in the control variables.

Individuals who are unemployed or economically inactive at the time of survey were treated like pensioners if they had an income in the past. In other words, their last observed income or occupation was used.

2.1.2. Individual income

Official registry data (e.g. from tax records) are most ideal to obtain high-accuracy measures of individual income. However, the linkage between genetic data and registry data was normally not feasible due to privacy concerns. Therefore, we mainly relied on self-reports of income, despite likely measurement error.

2.1.3. Household income

We considered household income as an alternative measure of individual income. Household income aggregates the individual incomes of all household members (e.g. spouses and possibly even children or other relatives). Therefore, household income captures not only factors that contribute towards individual income, but also other factors such as the ability and desire to attract a spouse and the characteristics of the spouse. Nonetheless, household income can still serve as a reliable proxy of individual income.

2.1.4. Occupational income

When detailed occupation information was available with standardized coding, we derived (the logarithm of) occupational income based on the national statistics data for each country. Occupation encompasses income potential and typically also reflects educational attainment, personal interests, social prestige and labor market opportunities. In comparison to individual income, occupational income only captures between-occupation variation in individual income. However, occupational income is less likely to suffer large measurement error because it is easier to recall occupation than income, while occupation-specific income is obtained from the national statistics of the relevant country. Occupational income measures were mainly used for larger cohorts. Due to different data availability across different countries in which those cohorts are based, slightly different approaches were used for different cohorts, which are summarized below.

2.1.4.1. UK Biobank and ALSPAC mothers

The UK Biobank recorded the occupation of participants with the UK's standardized occupational classification (SOC) 2000 version, which is coded in 4-digit numbers representing a hierarchical structure. Similarly, ALSPAC also provided occupational information in the same coding for the mother participants, while their income was not surveyed. For these British cohorts, we applied the approach that we developed in ref[1]. This approach was originally developed to impute income based on occupation and demographic information, rather than to derive occupational wage. The income imputed this way can be interpreted as expected income per occupation adjusted for demographics, which therefore is not essentially different from occupational income.

The details of the approach are available in the appendix of ref[1]. Here we only provide the overall summary. From the Annual Survey of Hours and Earnings, we obtained the tax-registry-based estimates of sex-specific mean and median hourly wages for each occupational group defined by 4-digit level SOC. Using the Labour Force Survey (LFS), a large representative survey data of the UK population, we fit a regression model of log hourly wages

using mean and median wages for each occupation along with demographic variables and interaction terms. The log occupational wages were then derived as the predicted outcomes from this regression. In the appendix of ref[1], it was shown that occupational wages constructed from this method yielded an out-of-sample R^2 = 0.50 with self-reported log hourly wages in British Household Panel Survey, another independent representative survey of the UK.

2.1.4.2. Lifelines and Netherlands Twin Registry

A similar approach was taken for two Dutch cohorts: Lifelines and Netherlands Twin Registry (NTR). We mirrored the approach for the British cohorts as closely as possible. Here we used data from the Dutch Labour Force Survey, 'Enquête Beroepsbevolking' (EBB). The EBB is a national representative survey of the Dutch labor force, conducted by Statistics Netherlands (CBS). We used a merged dataset containing 479,893 individuals in yearly waves from 2012 to 2017, where we excluded multiple observations per individual by taking the latest observation. The EBB used a Dutch version of standardized occupation codes, BRC, developed by CBS based on the International Standardized Classification of Occupation (ISCO) 08 standard.

As the EBB was the only national representative survey containing standardized occupation codes, we fitted a regression model and calculated the mean and median hourly wages per occupation group in the same sample. We standardized hourly wages to the year 2012 using the consumer price index calculated by CBS. We then calculated the mean and median wage for each 4-digit occupation code separately for each sex. If there are less than 10 people per occupation code, we calculated the mean and median using a pooled sample of both sexes. If there are less than 10 people per occupation code in the pooled sample, we used the 3-digit occupation code instead. If the 3 digit occupation code still did not yield a sufficient sample size, we used the 2-digit occupation code. The same model specification as the UK model was used for the wage prediction model.

Given the estimated model, we constructed the log hourly wages per occupation in the NTR and LifeLines. The accuracy of the model was tested by taking the 2017 EBB subset as a hold-out sample (N = 91,821) and re-estimating the regression model using the 2012 – 2016 subset excluding those present in the 2017 (N = 388,072). Regressing the log hourly wage on the imputed log hourly wage in the 2017 EBB subset yielded an R^2 of 0.47, which is similar to that for the UK case above.

2.1.4.3. Estonian Genome Center

For the Estonian Genome Center (EGCUT), we employed a simpler algorithm. We used the mean log wage of each occupation code, estimated for men and women separately, using the 2011 population census data from Statistics Estonia. EGCUT used 3-digit occupation codes based on the ISCO-88 standard while Statistics Estonia used occupation codes based on the ISCO-08 standard. The mean log wages for each ISCO-08 code were matched to the ISCO-88 codes based on the correspondence file published by the International Labour Organisation. When multiple ISCO-08 codes corresponded to a single ISCO-88 code, we took the average of the estimated means of the ISCO-08 codes.

We tested the accuracy of the occupational wage estimates by examining their correlation with the self-reported log wages in the Structure of Earnings Survey (*N*=369,247 individuals aged 25 to 64). This resulted in R^2 = 0.44, which is similar to the results of the Dutch and British cases.

2.1.4.4. HUNT

For the Norwegian cohort HUNT, we used a similar approach to that for EGCUT. Here, we used sex-specific mean wage statistics from 2015 to 2019 from the Statistics Norway (https://www.ssb.no/en/statbank/table/11418/). Similarly to the case of EGCUT, HUNT used 3-digit occupation codes based on the ISCO-88 standard while Statistics Norway used occupation codes based on the ISCO-08. The two are matched together in the same way as was done for EGCUT.

2.1.5. Parental income (iPSYCH)

While the income information of the participants of iPSYCH was available, they were too young that their current income was unlikely to reflect their life-time earnings potential. Therefore, we opted to use the income of their parents instead, which was collected from the Danish registry data. Specifically, we used the average earnings of the age 30 ~ 55 for each parent. This approach can be interpreted as using the offspring genotype as a proxy for the genotype of the parent.

2.2. Genotyping and imputation

Supplementary Table 3 reports cohort-level information on the genotyping platform, quality-control filters for the genotype data and subjects prior to imputation, subject-level exclusion criteria, and the reference panel and software used for imputation. As the reference panel for imputation, either the 1000 Genomes Project[2] or Haplotype Reference Consortium (HRC)[3] was used except for a few cohorts that additionally used cohort-specific reference data.

2.3. Association analyses

Each cohort estimated the following linear regression model for each SNP.

$$y_i = \beta_0 + \beta_1 SNP_i{}^j + Z_i{}'\gamma + \varepsilon_i$$

y_i is the log-transformed income phenotype for individual i, $SNP_i{}^j$ the count of effect-coded allele of the SNP j, Z_i the vector that contains control variables with corresponding coefficients γ, and ε_i the error component. Each cohort was asked to control for any sources of variation in income that do not reflect individual earning potential according to their data availability. This includes hours worked (with square and cubic terms), year of survey, indicators for employment status (retired, unemployed), self-employment, pension benefit, and etc (see Supplementary Table 4). Importantly, each cohort was asked to include at least top 15 genetic principal components (PC) to account for population stratification, as well as cohort-specific technical covariates related to genotyping (genotyping batches and platforms). For household income, the number of adult members was also controlled for if possible.

This model was estimated for male and female samples separately in light of the possible between-sex heterogeneity. Generally, the linear mixed model approach was preferred, which additionally models the error component with random genetic effects in order to account for the family structure and cryptic relatedness. The cohorts were advised to use BOLT-LMM[4] for implementation. For smaller family-based cohorts, for which BOLT-LMM's approximation approach was not expected to work well, fastGWA[5] was used instead. Otherwise, the association analysis was performed without the random effect component.

2.4. Quality control

We applied a stringent quality-control (QC) protocol to each set of GWAS results of each cohort based on the EasyQC software package (version 9.2) developed by the GIANT consortium[6], as well as additional steps developed by the SSGAC[7–9]. As the reference panel, we used HRC v.1.1[3]. All issues raised during the QC protocol were resolved through iterations with cohort analysts, before the meta-analyses.

The details of the QC protocols as well as the QC of the HRC reference panel is described in the supplementary materials of ref[9]. Here we only provide the overall summary. The main steps include removing SNPs with missing or incorrect numerical values (a *p*-value outside of [0,1], for instance); a minor allele frequency (MAF) below 0.1% or a minor-allele count (MAC) below 200; a low imputation accuracy (0.6 for MACH, 0.7 for IMPUTE, 0.8 for PLINK); the effect-coded allele or the other allele with values different from "A," "C," "G," or "T."; a Hardy-Weinberg Equilibrium *p*-value lower than 10^{-3} ($N<1000$), 10^{-4} ($1000 \leq N < 2000$), or 10^{-5} ($2000 \leq N < 10000$) ; and an allele frequency different from the allele frequency in the reference panel by more than 0.2. We also removed duplicate SNPs or SNPs absent in the reference panel.

After applying these steps, the resulting output was inspected to determine if an unusual number of SNPs were removed during one of the steps and when necessary errors were resolved together with the cohort analysts.

2.5. Meta-analysis

To obtain a unified GWAS output that integrates multiple GWAS results across different income measures collected from various cohorts, we conducted a multi-step meta-analysis process as follows:

2.5.1. Step 1: Sex-Stratified Meta-Analysis Using METAL

We began by conducting meta-analyses separately for each income measure and sex. For this, we used METAL with sample-size weighting to combine cohort-level GWAS results, resulting in eight sets of GWAS summary statistics (four income measures for each sex). We opted for METAL at this stage because there was no expectation of sample overlap across different cohorts.

2.5.2. Step 2: Combining Male and Female Results with MTAG

Next, for each income measure, we combined the male and female meta-analysis results using MTAG, specifying perfect genetic correlation and equal heritability between sexes. This configuration in MTAG functions similarly to a generalized inverse-variance-weighted meta-analysis, using information from the intercepts of LD score regressions to calculate weights and standard errors. This approach helped address potential relatedness between the male and female samples, which METAL could not account for. Prior to running MTAG, we excluded SNPs with a combined sample size ($N = N_{male} + N_{female}$) less than 50% of the maximum to ensure that no SNPs had an excessively small sample size.

2.5.3. Step 3: Combining Different Income Measures with MTAG

To integrate the four income measures, we again used MTAG, allowing for different heritability estimates across the measures. This adjustment accounted for potential variability in measurement error among the income measures, making MTAG more efficient than METAL in this context. Accounting for sample overlap was critical here, as some individuals were included in multiple GWAS analyses involving different income measures. For example, the UKB sample had household and occupational income measures, resulting in overlapping samples between both GWAS.

Unlike METAL, MTAG can only output SNPs common across all input GWAS summary statistics, which resulted in a reduced number of SNPs (4,885,528) due to limited genome coverage in the individual and parental income GWAS results. To address this limitation, we repeated Step 3 while excluding 1) individual income, 2) parental income, and 3) both. We found that all four sets of results, including the full set, exhibited pairwise genetic correlations above 0.99 and nearly identical heritability estimates, indicating that the results were not sensitive to excluding individual or parental income. We then selected the SNP with the largest absolute Z statistic from the four sets for each available SNP.

2.5.4. Step 4: Final SNP Selection and Effective Sample Size Calculation

After these analyses, we obtained 4,885,528 SNPs from the MTAG that included all measures and 6,599,628 SNPs from the MTAG with only occupational and household income. We excluded 2,353,649 SNPs with an effective sample size below 70% of the maximum effective sample size (692,936), resulting in a final set of 9,131,507 SNPs.

2.5.5. Evaluation of Meta-Analysis Results

We observed that the MTAG results using perfect genetic correlation closely matched those obtained from genomic SEM's common factor model, with a correlation (R^2) of 0.998 between the Z statistics from both methods. The mean χ^2 statistic was marginally higher for MTAG (2.118 vs. 2.108), leading us to define the meta-analyzed outcome as the "Income Factor" to emphasize the shared genetic factor across different income measures.

2.5.6. Effective Sample Size Estimation

We estimated the effective sample size per SNP based on standardized beta estimates ($Z/\sqrt{N}$ for large *N*). Using MTAG's standardized estimates, we computed the effective sample size as

$$N_{eff} = (\frac{Z}{\beta_{std}})^2.$$

For downstream analyses, we averaged these per-SNP effective sample sizes for SNPs with a minor allele frequency (*MAF*) between 0.1 and 0.4 to reduce noise. This yielded an estimated overall sample size of 668,288 for the Income Factor GWAS.

2.5.7. Bias Correction and Effect Size Measurement

MTAG inherently applies bias correction using the intercept of the LD score regression, so no additional bias adjustments were made. We measured effect sizes using the (partial) coefficient of determination (R^2), calculated as the square of the standardized beta estimates.

2.6. Identification of genomic loci

We used FUMA v1.5.2[13] with the default parameters to define genomic loci associated with Income Factor. Here, we briefly explain the procedure. See the original paper for more detail. FUMA first finds independent significant SNPs with a genome-wide significance ($p < 5x10^{-8}$) such that they are independent from each other at $r^2 < 0.6$. Then, independent lead SNPs are identified from the independent significant SNPs such that they are independent from each other at $r^2 < 0.1$. FUMA then forms genomic loci by grouping independent significant SNPs if they are less apart than 250 kb. As a result, a genomic risk locus can contain multiple independent significant SNPs and multiple lead SNPs. To define the border of each locus, SNPs that have $r^2 \geq 0.6$ are identified for each of the independent significant SNPs from the reference data, including those not available in the input GWAS summary statistics. We used the 1000 Genomes Project as the reference data, which is readily available in FUMA.

In addition, we ran Conditional and Joint Association Analysis (COJO) using the Genome-wide Complex Trait Analysis (GCTA) software to refine our understanding of the genetic architecture underlying the trait of interest (J. Yang et al., 2012). The analysis was performed with a window size of 100,000 base pairs (bp), conditioned on 207 primary lead SNPs from 162 loci, previously identified as significantly associated with the Income Factor. Our COJO analysis revealed 57 secondary lead SNPs that surpassed the Bonferroni corrected threshold for statistical significance ($p <= 5\times10^{-8}$), conditioning on the primary lead SNPs. Notably, 55 of these secondary lead SNPs were located within the original primary genomic loci, underscoring their potential role in the same genetic regions initially implicated in the association with the Income Factor. The remaining two secondary lead SNPs were identified at novel loci, which had not been recognized in relation to the primary lead SNPs. These findings highlight the presence of additional genetic variants that contribute to the Income

factor, thereby enriching our understanding of its complex genetic basis. See details of these secondary lead SNPs in Supplementary Table 30.

2.7. Winner's-curse adjustment

We used an empirical Bayes framework to adjust for winner's curse bias in the estimated effect sizes from the lead SNPs, following the approach described in ref[7,14]. The marginal effect sizes of SNPs are assumed to be drawn from the following mixture distribution: $N(0, \tau^2)$ with probability π and 0 otherwise. Here π is the fraction of the non-null SNPs and τ^2 is their effect-size variance.

We estimated the parameters π and τ^2 by maximum likelihood, using all the SNPs from our Income Factor meta analysis, which yielded $\hat{\pi}$ = 0.65 and $\hat{\tau}^2$ = 3.01×10^{-6}. On average among the SNPs with 0.1 < MAF < 0.4, this corresponds to the shrinking factor of 0.67, which implies that we need to shrink the GWAS effect estimate by 33% to obtain the winner's-curse-adjusted estimate, conditioning that the SNP is non-null. For full technical details of these derivations, see the Supplementary Note of ref[7,14].

We then computed the 5th, 50th and 95th percentile of the effect-size distribution of the lead SNPs as follows. We simulated 10,000 effect sizes from the posterior distribution of each lead SNP and obtained the 5th, 50th and 95th percentiles from the complete set of simulated effect sizes.

3. Environmental heterogeneity

We investigated the potential environmental heterogeneity in the GWAS of income by examining the cross-cohort genetic correlations by sex or by country.

3.1. Between-sex heterogeneity

We estimated genetic correlation using LDSC between male and female meta-analysis results for each income measure (from Step 1 in Section 2.5). In addition, we conducted Income Factor GWAS on the sex-specific results (Step 3 in Section 2.5), which yielded an effective sample size of 360,196.7 for men and 353,429.1 for women. We then estimated the genetic correlation between the sex-specific Income Factor results.

3.2. Cross-country heterogeneity

We derived country specific GWAS meta-analyses on two measures of income, occupational and household income, for which we were able to secure a sufficiently large sample size for multiple countries. We applied Step 1 and 2 in Section 2.5 using the cohorts from each country. As a result, we obtained the household income GWAS for the USA (N_{eff} = 30.855), the UK (N_{eff} = 387,579), and the Netherlands (N_{eff} = 40,533); and the occupational income GWAS for Estonia (N_{eff} = 75,682), Norway (N_{eff} = 42,204), the UK (N_{eff} = 279,883), and the Netherlands

(N_{eff} = 24,425). We then estimated pairwise gene`tic correlations between these results with LDSC.

Next, we examined whether our meta-analysis results could be driven by the cohorts from the UK due to a dominantly large share of the British cohorts in the meta-analysis. We repeated the meta-analysis procedure (Step 1-3 in Section 2.5) separately with the British and non-British cohorts (from 11 countries), which yielded two GWAS results for Income Factor. The effective sample size was 414,978 for the UK result and 330,639 for the non-UK result. We then estimated the genetic correlation between them.

4. Comparison with educational attainment

We compared our Income Factor GWAS results with the GWAS of educational attainment (EA, measured as years of education) in several approaches by examining 1) implicated genes and biological functions, 2) genetic correlation with LDSC, 3) polygenic overlap with MiXeR[15], and 4) GWAS-by-subtraction[16].

Here, we used a version of EA summary statistics that is slightly different from those publicly available. The latest EA GWAS study[17] revised the coding of the years of schooling in the UKB, which better reflects the educational qualification of the participants. We conducted a GWAS of EA in the UKB based on the new coding. Then, by using MTAG with the meta-analysis option, we meta-analyzed the UKB result with EA3 summary statistics that did not include the UKB. This increased the mean χ^2 from 2.53 of the original EA3 result to 2.94. We found 872 loci tagged by 1,473 lead SNPs.

4.1. Biological annotation

To examine if the Income Factor was capturing the same underlying biology as household income and educational attainment, we used MAGMA and a test of overrepresentation performed using the GENE2FUNC process in FUMA (version 1.5.2). First, gene-based statistics were derived for the Income Factor and educational attainment (Lee et al. 2018) using MAGMA. For household income, the MAGMA gene-based statistics were taken from Supplementary Table 18 by Hill et al. (2019). Next, genes that passed a Bonferroni correction were retained and compared across the Income Factor, educational attainment (Lee et al. 2018), and household income (Hill et al. 2019). This comparison can be seen in the Venn diagram in Extended Fig 7a.

Second, using the GENE2FUNC in FUMA, we performed a hypergeometric test to determine if the genes identified using MAGMA were overrepresented in biological pathways using MsigDB. Gene sets that attained statistical significance (FDR <0.05) in the Income Factor, educational attainment, and household income were retained and compared against each other (Extended Fig 7b).

4.2. LDSC and MiXeR

Using LDSC[10], we estimated that the genetic correlation between Income Factor and EA is 0.92 (*s.e.* = 0.01). This result was consistent with the previous reports, which ranged from 0.90 to 0.94[1,18,19]. Though providing a useful summary of the shared genetic basis, the global genetic correlation only estimates the average correlation of genetic associations and does not capture mixtures of effect directions.

To gain further insights, we used MiXeR[15] tool to estimate the degree of polygenic overlap between the Income Factor and EA. MiXeR exploits a bivariate causal mixture model, which allows for estimating: 1) the number of non-null SNPs specific to each trait, 2) the number of SNPs with non-null effect for both traits, and 3) the genetic correlation within the shared variants.

More specifically, MiXeR models GWAS effects as a mixture of four components: *1)* SNPs with null effects for both traits, *2)* non-null SNPs specific to trait 1, *3)* non-null SNPs specific to trait 2, and *4)* SNPs with non-null effects for both traits. Each of the four components is represented by the proportion of its member SNPs denoted by π_o, π_1, π_2, and π_{12}, respectively. The SNPs in the second and third components (non-null SNPs specific to each trait) are assumed to be distributed with $N(0, {\sigma_k}^2)$ for trait k = 1 and 2, respectively. The SNPs in the fourth component (the shared non-null SNPs) are distributed with a bivariate normal distribution with the variance-covariance matrix:

$$\begin{bmatrix} {\sigma_1}^2 & \rho_{12}{\sigma_1}^2{\sigma_1}^2 \\ \rho_{12}{\sigma_1}^2{\sigma_1}^2 & {\sigma_2}^2 \end{bmatrix}$$

where ρ_{12} indicates the correlation of the GWAS effects within the shared SNPs.

Under the MiXeR model, the global genetic correlation is estimated as: $\rho_{12}\pi_{12} / \sqrt{(\pi_1 + \pi_{12})(\pi_2 + \pi_{12})}$, which is the correlation of effects within the shared variants scaled by the normalized degree of their polygenic overlap. Therefore, a low global genetic correlation may indicate a low correlation within the shared variants and/or a low degree of polygenic overlap.

The estimated model suggests that the set of SNPs associated with the Income Factor is entirely nested within the set of SNPs associated with EA, with 83.2% of the EA SNPs shared with income (**Extended Data Fig. 3a**). Furthermore, the genetic correlation within the shared component is perfect (r_g = 1.00, s.e. = 0.002). As a result, the global genetic correlation, which is a composite measure of the polygenic overlap and genetic correlation within the shared set, was estimated to be 0.91 (s.e. = 0.01), consistent with the LDSC result.

4.3. GWAS-by-subtraction

To explore the results from MiXeR, we statistically decomposed the estimated genetic association of Income Factor into the indirect effect due to EA and the direct effect unexplained by EA (denoted 'NonEA-Income' hereafter), using the GWAS-by-subtraction approach[16]. While this method was implemented as a Cholesky model in the original study, we implemented this in a form of the mediation model, which produces numerically equivalent results. The main difference is that we did not specify latent factors whose variance was fixed

to unity. This therefore only affects the scale of the beta estimates and standard errors, while the Z statistics are the same.

More specifically, we set up a genetic mediation model of Income Factor with EA as an mediator (**Extended Data Fig. 3b**). Under this model, the genetic association of Income Factor for SNP j (${\beta_j}^{INC}$) can be written as ${\beta_j}^{INC} = \alpha \times {\beta_j}^{EA} + \delta_j$, where each component is defined as:

- $\alpha \times {\beta_j}^{EA}$: indirect mediated effect that captures the genetic association of EA (${\beta_j}^{EA}$) scaled by the correlation between Income Factor and EA (α)
- δ_j: direct effect representing the genetic association of Income Factor unexplained by EA (NonEA-Income).

In this model, the SNPs that are associated with EA but not associated with income (corresponding to the blue part in **Extended Data Fig. 3a**) are SNPs whose direct effects on income (δ_j) are strong enough to offset their indirect effects ($\alpha \times {\beta_j}^{EA}$). These are SNPs with effects whose signs are discordant across EA and NonEA-Income. On the other hand, SNPs with null association for NonEA-Income imply that there will be a perfect genetic correlation between income and EA within such SNPs (corresponding to the orange part of **Extended Data Fig. 3a**). It is important to note that this model does not imply any causal direction between the variables. The above decomposition always holds for any given GWAS effects from two traits.

We estimated this model using genomic SEM[11], which essentially involves estimating the non-EA genetic association of Income Factor (δ_j) and the correlation between EA to income (α).

Instead of the default European reference panel from phase 3 of the 1000 Genomes Project[2] provided by genomic SEM, we used the HRC European reference panel to increase the SNP coverage. Genomic SEM uses a reference panel to align SNPs and obtain MAF estimates, which in turn are used to compute the per-allele effect sizes standardized with respect to the phenotype. As a result, 7,274,585 SNPs were included in the final output.

4.4. Concordant and discordant sets

We classified the SNPs as concordant or discordant on the basis of the sign concordance of $\hat{\delta}_j$ and ${\hat{\beta}_j}^{EA}$ estimates. Out of 7,274,585 SNPs, 4,056.295 SNPs were classified as discordant (corresponding to 55.8%). The sign discordance here implies that the size of ${\hat{\beta}_j}^{INC}$ is much smaller than that of ${\hat{\beta}_j}^{EA}$.

To validate this result, we tested in an independent sample whether the SNPs with the sign discordance between EA and NonEA-Income had weaker genetic associations of income. We grouped the SNPs into two sets according to the sign concordance. We then estimated the partitioned heritability of income for these two groups of SNPs in unrelated individuals from the sibling subsample of the UKB.

More specifically, we used GCTA[20] to construct two genomic relatedness matrices (GRM), one with 681,049 discordant SNPs and the other with 537,607 concordant SNPs, all

of which were from the HapMap3 set. Here the SNPs were stratified from the GWAS results excluding the UKB sibling sample and their close relatives. After removing the individuals whose occupational or household income was not available, we identified unrelated individuals from the sibling sample by applying *--grm-singleton 0.05*, using a merged GRM from the two GRMs. Then, the heritability was estimated each for occupational and household income, specifying the two GRMs separately in the model. The sample size was 12,689 and 16,972 for occupational and household income, respectively. As covariates, we included age, age^2, age^3, sex, dummies for survey year, and interactions between sex and the rest.

We confirmed that the heritability of income was contributed disproportionately more by the concordant SNPs and far less by the discordant SNPs. The concordant SNPs accounted for 70.0% of the heritability (*s.e.* = 19.5%) for occupational income and 75.3% for household income (*s.e.* = 15.8%). In both cases, only the heritability component for the concordant SNPs was statistically significant.

5. Polygenic score analyses of income

5.1. Baseline polygenic prediction

We conducted a validation analysis based on polygenic prediction with 1KG-EUR-like individuals in the Swedish Twin Registry (STR), which was not included in our meta-analysis. We chose the STR as the main prediction cohort for its accurate income data collected from administrative data sources, which include individual, occupational, and household income (see the subsection below for the detail). In addition, we also used the UKB siblings (UKB-sib) and the Health and Retirement Study (HRS) from the US as prediction cohorts. For the UKB-sib, occupational and household income measures were available, while a self-reported individual income measure was available for the HRS.

We constructed polygenic indexes (PGI), using the meta-analysis results of income excluding a prediction cohort at a time, as well as a PGI based on the EA GWAS summary statistics in the same way for comparison. PGIs were created only with HapMap3 SNPs[12] as these SNPs are known to have good imputation quality and provide good coverage in 1KG-EUR-like samples. Furthermore, the SNPs were limited to those available in both income and EA summary statistics for the sake of precise comparison. We used the reference panel from the HRC. The details of QC for this panel can be found in the Supplementary Information of ref[17].

We derived PGIs based on a Bayesian approach implemented in the software LDpred2[21]. LDpred2 is an extension of LDpred[22], which adjusts for LD and computes individual SNP weights by using posterior means of LD-independent effect-size distributions. LDpred2 improves LDpred approach by 1) using a LD window based on genetic distances, which can better accommodate long LD regions and 2) allowing for Bayesian updating of p (the proportion of causal SNPs) and h^2 (SNP heritability) parameters (called LDpred2-auto). As priors, we set 0.2 for p and LDSC h^2 estimates for h^2 parameters. While the authors of LDpred2 recommend running LDpred2 genome-wide, we ran Ldpred2 per chromosome for its

computational efficiency given that prediction results are barely different for a well-powered GWAS.

Since the STR sample was genotyped with three different platforms, which gave too few common HapMap3 SNPs after quality-control filters, we applied LDpred2 for the SNPs available in each batch and created PGIs for each batch. We then included indicators for these different batches in the prediction analyses.

In order to create PGIs for the UKB siblings, we re-conducted the GWAS of income and EA excluding the sibling sample as well as their close relatives (up to the third degree of relatedness). We then performed the meta analyses again.

For both the STR and the UKB siblings, we randomly chose one sibling from each family to avoid complications due to having relatives in the sample. We measured the prediction accuracy on the basis of the incremental R^2, which is the difference between the R^2 from a regression of the phenotype on the PGI and the baseline covariates and the R^2 from a regression on the baseline covariates only. We constructed confidence intervals for the incremental R^2 by bootstrapping the sample 1,000 times.

Because income typically contains substantial demographic variation, we pre-residualized the log of income for demographic covariates. Then, as baseline covariates, we only included top 20 genetic PCs and genotype batch indicators. Because income data was available for multiple years for the STR and the HRS, we residualized the log of income for age, age^2, age^3, sex, and interactions between sex and the age terms within each year and obtained the mean of residuals for each individual. For the UKB-sib, which only had cross-sectional data, we residualized the log of income for age, age^2, age^3, sex, dummies for survey year, and interactions between sex and the rest. For EA measure (years of education), we applied the same procedure for consistency while using dummies of birth year in place of the age terms.

5.1.1. The STR income data description

Individual income

We used work income (including work-related benefits) for each year from 1990 to 2018 from Sweden's registry data. For the years of 1970, 1975 and 1985, the census data was also used. The sample was limited to individuals of at least 30 years of age at each point. A few extreme outliers were removed by trimming at the 99th percentile.

Household income

Household income data only existed for a few years in the late census data. Therefore, all the observations were taken from the 1990s. The same sample filtering and the outlier removal was applied.

Occupational income

Work income averages (including work-related benefits) per category of three-digit ISCO codes (*SSYK*, the Swedish version) were obtained per year from 2001 to 2016 from the population level data (except for 2014 where the occupation codes were missing). These were then matched to the corresponding year-code observations in the STR data.

5.2. Within-family polygenic prediction

Genetic associations of SES traits are known to particularly suffer confounds due to indirect genetic effects such as genetic nurture and population stratification[23–25]. Recent studies[17,26] have shown that, by controlling for parental PGIs, the direct genetic effect can be isolated from the overall population effect captured by the PGI. In the case of EA, the direct genetic effect was shown to account for 30.9% of the overall predictive power of the EA PGI.

We followed the same approach to estimate the share of the direct genetic effect in the overall population effect captured by Income Factor PGI. We imputed missing parental genotypes from sibling and parent-offspring pairs, using the tool *snipar*[27]. To apply the imputation algorithm, we prepared the data as follows. We first identified individuals with "White British' ancestry" and first-degree relatives based on the kinship coefficients (first-degree if > 0.177) provided by the UKB. We then used KING software[27] to infer the sibling and parent–offspring relations by specifying "--related-degree 1".

As inputs for snipar, we only used high quality common SNPs from the HapMap3 set as well as directly genotyped SNPs (imputation INFO score > 0.99 and MAF>1%). We first phased these SNPs from the imputed genotype data using eagle v2.4.1[28] with 1000 Genomes Phase 3 reference panel. We then used these phased SNPs as inputs for snipar to impute missing parental genotypes. This procedure resulted in 1,244,153 SNPs in total whose missing rate was lower than 1%. The average observed proportion of IBD0 pairs was 22.3%, which closely matched the expected proportion of 22.5% given that the sibling pairs were the majority. The estimated genotyping error was also sufficiently low, with an average of 0.07%.

Following the same procedure in Section 5.1, we created a PGI for Income Factor by applying LDpred2 to this set of SNPs for the UKB sibling sample. For each individual in this sample, we also created parental PGIs using the imputed parental genotypes. Each PGI was then normalized to have a variance of 1. The phenotypes (the log of occupational income or household income) were residualized for age, age^2, age^3, sex, dummies for survey year, and interactions between sex and the age and year terms, as well as top 20 genetic PCs and genotype batch indicators. Then, the residualized phenotypes were also normalized to have a variance of 1 separately in males and females. As a result, regression estimates of PGI represent (partial) correlations, and their squares indicate proportions of phenotypic variance explained.

For the prediction analysis, we estimated the following regression model:

$$y_{ij} = \delta PGI_{ij} + \alpha(PGI_{p(j)} + PGI_{m(j)}) + \varepsilon_{ij}$$

where y_{ij} is the phenotype of individual i in family j, PGI_{ij} the PGI, $PGI_{p(j)}$ the paternal PGI, $PGI_{m(j)}$ the maternal PGI, and ε_{ij} the error term. δ represents the direct genetic effect of the PGI and α reflects indirect genetic effects and the effects of other genetic and environmental factors including the confounding due to non-random mating. The derivations in ref[17,26] show that this regression gives an unbiased estimate of δ, while the population effect, denoted ψ, is then equal to $\delta + (1 + r_{am})\alpha$, where r_{am} is the correlation between $PGI_{p(j)}$ and $PGI_{m(j)}$.

We fitted the above regression by OLS and clustered the standard errors by family to account for within-family dependence. We estimated r_{am} using the correlation between siblings' PGI, which is equal to $(1 + r_{am})/2$ (see the supplementary note section 8 of ref[26]). For Income Factor PGI, this was estimated to be 0.107. Given these estimates, we computed

estimates for ψ as well as the ratio of direct genetic effect to the population effect, δ / ψ . We then derived standard errors for the estimates of ψ and δ / ψ by using the delta method.

5.3 Polygenic prediction in samples of non-European descent

We performed polygenic prediction analysis on the non-EU samples in the UK Biobank. The UK Biobank has a self-reported ethnic background data field (21000), from which we extracted samples that self-reported as African, Caribbean, or Indian. These three groups have the largest sample sizes among the non-EUR ancestry groups, which contain $N_{African}$ = 3,210, $N_{Caribbean}$ = 4,300, and N_{Indian} = 5,661 samples, respectively. Furthermore, we also used genetic ancestry groups derived from SNP data, which tends to be more accurate. In this latter approach, we also included the three largest sub-samples ($N_{Africans}$ = 9,494, East-Asians $N_{East\text{-}Asians}$ = 2,216, and $N_{South\text{-}Asian}$ = 11,413).

To derive different ancestry groups from the genetic data, we first converted the imputed genotype dosages for HapMap3 SNPs into hard calls. Then, we merged the data with all samples from the third phase of the 1000 Genomes Project. We dropped SNPs that had a call rate less than 99% or minor allele frequency less than 1% in the merged sample. We computed the PCs using the 1000 Genomes sample, projected the remaining individuals onto this PC space. We identified an individual as having EAS, SAS, or AFR genetic ancestry if they are within four standard deviations from the mean of that PC in the respective 1000 Genomes sample for each of the first ten PCs.

To derive polygenic scores for non-EU samples, we first calibrated the estimated effect sizes of the income factor GWAS using LDpred2 across the aforementioned non-European ancestry groups. The calibration aimed to refine the effect size estimates for these population groups. Next, we constructed polygenic indexes (PGIs) using the calibrated effect sizes of the HapMap3 SNPs, following the protocol described in Section 5.1.

For each ancestry group, the linkage disequilibrium (LD) matrix was estimated based on the genotypes of the predicted samples to ensure that the LD structure reflected the specific genetic makeup of the target samples. We then proceeded with regression analysis to examine the associations between the polygenic indexes and income outcomes, using the same approach and co-variates as in the other UKB prediction analyses (see Section 5.1).

Results for self-reported ethnic background groups: For household income, the predictive accuracy of the PGI for African, Caribbean, and Indian ancestry groups were ΔR^2 = 0.40% (95% CI: 0.00% - 0.73%), ΔR^2 = 0.64% (95% CI: 0.08% - 1.06%), and ΔR^2 = 1.62% (95% CI: 0.94% - 2.33%), respectively. For occupational income, the predictive accuracy of the corresponding PGI were ΔR^2 = 0.85% (95% CI: 0.10% - 1.44%), ΔR^2 = 0.16% (95% CI: 0.00% - 0.32%), and ΔR^2 = 1.86% (95% CI: 1.13% - 2.57%), for African, Caribbean, and Indian ancestry groups, respectively.

Results for genetic ancestry groups: For household income, the predictive accuracy of the PGI for African, East Asian, and South Asian ancestry groups were ΔR^2 = 0.38% (95% CI: 0.07% - 0.61%), ΔR^2 = 0.09% (95% CI: 0.00% - 0.17%), and ΔR^2 = 1.58% (95% CI: 1.05% - 2.06%), respectively. For occupational income, the predictive accuracy of the corresponding PGI were ΔR^2 = 0.45% (95% CI: 0.10% - 0.71%) for African group, ΔR^2 = 0.16% (95% CI:

0.00% - 0.32%) for East Asian group, and ΔR^2 = 1.34% (95% CI: 0.85% - 1.84%) for South Asian group.

Thus, polygenic indices derived from GWAS results on European samples have substantially reduced (but non-zero) predictive accuracy in non-European populations.

6. Genetic correlation analysis

We estimated genetic correlations of Income Factor, EA, and NonEA-Income with a wide set of traits, including socioeconomic, behavioral, and physical and mental health traits. We used LDSC to estimate the genetic correlations (r_g), using the pre-computed LD scores from the authors. and computed the difference in r_g between EA and NonEA-Income. We derived the standard errors for the difference in r_g on the basis of jackknife estimates, using the same default approach of LDSC. Then, the false discovery rate correction was applied to the resulting p-values.

The phenotypes included in this genetic correlation analysis are as follows: Subjective well-being[14], Parental lifespan[29], Cognitive performance[7], General risk tolerance[9], Chronotype[30], Sleep duration[30], Age of smoking initiation[31], Smoking persistence[31], Cigarettes per day[31], Drinks per week[31], Alcohol dependence[32], Height[33], BMI[33], Waist-to-hip ratio[33], Blood pressure (in-house GWAS conducted in the UKB sample), Type 2 diabetes[34], Triglycerides[35], ADHD[36], Bipolar disorder[37], Schizophrenia[38], Autism spectrum[39], Anorexia nervosa[40], Obsessive compulsive disorder[41], Major depressive disorder[42], Anxiety disorder[43], Neuroticism[44], Stress-related disorder[45], Cannabis use disorder[46], Cross disorder[47]

7. Biological annotation

7.1. Gene mapping

We used FUMA v1.5.2[13] with the default parameters to find genes implicated in Income Factor GWAS, which used four mapping approaches to link the identified SNPs to protein-coding genes. First, the genes were mapped to the SNPs on the basis of physical proximity with a 10 kb window. Second, the genes were mapped according to expression quantitative trait locus (eQTL). We used the eQTL data from GTEx v8[48] and BRAINEAC[49]. Third, the genes were mapped based on significant chromatic interactions with the builtin chromatic interaction data. Fourth, we considered the genes that were statistically significant with Bonferroni correction from MAGMA gene-based association tests, which convert the mean chi-square of member SNPs into a gene-level test statistic.

7.2. Tissue-specific enrichment analysis

We performed tissue-specific enrichment analyses using two approaches: LDSC-SEG[50] and MAGMA gene-property analyses[51]. First, we applied LDSC-SEG to estimate tissue or cell-type specific enrichment, using the pre-computed LD scores by the authors according to the gene expression annotations from Franke lab[52] and GTEx v6 data[48]. Second, MAGMA gene-

property analysis was used to examine relationships between gene-level associations and tissue-specific gene expression profiles. The gene expression data were taken from GTEx v8.

8. GREML heritability estimation

We estimated the heritability of different income measures in the STR and UKB-sib samples with GREML from GCTA[20]. Such estimates are useful for gauging the maximum predictive power that could be achieved by the PGI. For both data sets, only the HapMap3 SNPs were included in the GRM and unrelated individuals were identified by applying *--grm-singleton 0.05*. The phenotypes were residualized (and averaged per individual for the STR) prior to the analyses in the same way from the prediction analyses. The results were reported in the **Supplementary Table 13** along with the LDSC heritability estimates from the GWAS meta-analysis results.

9. Phenome-wide association study

We explored the clinical relevance of the Income Factor PGI for common diseases in the sibling sample of the UKB. We conducted a phenome-wide association study, using the in-patient electronic health records for 115 diseases with sex-specific sample prevalence no lower than 1%. We derived case-control status according to the phecode scheme by mapping the UKB's ICD-9/10 records to phecodes (https://phewascatalog.org/phecodes, version 1.2)[53,54]. These ICD-9/10 records were collected from hospitalization, cancer, and death registries (as of May 2021).

We fitted a linear regression of case-control status on the Income Factor PGI while controlling for the parental PGIs to specifically capture the direct genetic effects of income PGI. As covariates, we also included year of birth, its square term, and their interactions with sex, genotype batch dummies, and 20 genetic PCs. The standard errors were clustered by family.

In total, 14 diseases from various categories were significantly associated with the direct genetic effect of the Income Factor PGI at false discovery rate < 0.05 (**Extended Fig. 5** and **Supplementary Table 27**). The results suggest that having a higher Income Factor PGI can lead to lower risk for cardiovascular diseases, digestive issues, Type 2 diabetes, obesity, depression, tobacco use disorder, and musculoskeletal issues.

10. Cohort acknowledgements

10.1. ALSPAC (Avon Longitudinal Study of Parents and Children)

We are extremely grateful to all the families who took part in this study, the midwives for their help in recruiting them, and the whole ALSPAC team, which includes interviewers, computer and laboratory technicians, clerical workers, research scientists, volunteers, managers, receptionists and nurses. The UK Medical Research Council and Wellcome (Grant ref: 217065/Z/19/Z) and the University of Bristol provide core support for ALSPAC. This publication is the work of the authors and they will serve as guarantors for the contents of this

paper. GWAS data was generated by Sample Logistics and Genotyping Facilities at Wellcome Sanger Institute and LabCorp (Laboratory Corporation of America) using support from 23andMe.

Please note that the study website contains details of all the data that is available through a fully searchable data dictionary and variable search tool" and reference the following webpage: http://www.bristol.ac.uk/alspac/researchers/our-data/. Ethical approval for the study was obtained from the ALSPAC Ethics and Law Committee and the Local Research Ethics Committees. Consent for biological samples has been collected in accordance with the Human Tissue Act (2004). Informed consent for the use of data collected via questionnaires and clinics was obtained from participants following the recommendations of the ALSPAC Ethics and Law Committee at the time. At age 18, study children were sent 'fair processing' materials describing ALSPAC's intended use of their health and administrative records and were given clear means to consent or object via a written form. Data were not extracted for participants who objected, or who were not sent fair processing materials.

Study data were collected and managed using REDCap electronic data capture tools hosted at the University of Bristol. REDCap (Research Electronic Data Capture) is a secure, web-based software platform designed to support data capture for research studies. DOI: 10.1016/j.jbi.2008.08.010

Pregnant women resident in Avon, UK with expected dates of delivery between 1st April 1991 and 31st December 1992 were invited to take part in the study. 20,248 pregnancies have been identified as being eligible and the initial number of pregnancies enrolled was 14,541. Of the initial pregnancies, there was a total of 14,676 foetuses, resulting in 14,062 live births and 13,988 children who were alive at 1 year of age. The total sample size for analyses using any data collected after the age of seven is therefore 15,447 pregnancies. Of these 14,901 children were alive at 1 year of age. Of the original 14,541 initial pregnancies, 338 were from a woman who had already enrolled with a previous pregnancy, meaning 14,203 unique mothers were initially enrolled in the study. As a result of the additional phases of recruitment, a further 630 women who did not enrol originally have provided data since their child was 7 years of age. This provides a total of 14,833 unique women (G0 mothers) enrolled in ALSPAC as of September 2021. G0 partners were invited to complete questionnaires by the mothers at the start of the study and they were not formally enrolled at that time. 12,113 G0 partners have been in contact with the study by providing data and/or formally enrolling when this started in 2010. 3,807 G0 partners are currently enrolled.

10.2. CoLaus (Cohorte Lausannoise)

The authors would like to thank all the people who participated in the recruitment of the participants, data collection and validation, particularly Nicole Bonvin, Yolande Barreau, Mathieu Firmann, François Bastardot, Julien Vaucher, Panagiotis Antiochos, Cédric Gubelmann, Marylène Bay and Benoît Delabays.

10.3. Croatia - Korcula

This research was funded by the Medical Research Council UK, the Croatian National Centre of Research Excellence in Personalized Healthcare grant (number KK.01.1.1.01.0010), and the Centre of Competence in Molecular Diagnostics (KK.01.2.2.03.0006).

10.4. EGCUT (Estonian Genome Center, University of Tartu)

The authors wish to acknowledge the participants of the Estonian Biobank for their contributions.
The activities of the EstBB are regulated by the Human Genes Research Act, which was adopted in 2000 specifically for the operations of EstBB. Individual level data analysis in EstBB was carried out under ethical approval by the Research Ethics Committee of the University of Tartu (Approval number 288/M-18), using data according to release application 6-7/GI/33516 from the Estonian Biobank.

The Estonian Genome Center analyses were partially carried out in the High Performance Computing Center, University of Tartu. The authors also acknowledge support for the development of the infrastructure of the Estonian Genome Centre from the Estonian Research Infrastructures Roadmap project No. SP1GI16442T "Estonian Centre for Genomics II". The work of the Estonian Genome Center, University of Tartu was funded by the European Union through Horizon 2020 research and innovation program under grants no. 810645 and 894987, through the European Regional Development Fund projects GENTRANSMED (2014-2020.4.01.15-0012), MOBEC008, MOBERA21 and Estonian Research Council Grants PRG791 and PRG1291. We also acknowledge the Estonian Biobank Research Team (E-mail: EstBBresearch@ut.ee) responsible for data collection, genotyping, quality control and imputation, including Andres Metspalu (andres.metspalu@ut.ee), Lili Milani (lili.milani@ut.ee), Reedik Mägi (reedik.magi@ut.ee), Mari Nelis (mari.nelis@ut.ee), Georgi Hudjashov (georgi.hudjashov@ut.ee).

10.5. FTC (Finnish Twin Cohort)

Phenotype and genotype data collection in the twin cohort has been supported by the Wellcome Trust Sanger Institute, the Broad Institute, ENGAGE – European Network for Genetic and Genomic Epidemiology, FP7-HEALTH-F4-2007, grant agreement number 201413, and the Academy of Finland (grants 264146, 308248, 312073, 336823, and 352792 to JKaprio).

10.6. HUNT (Trøndelag Health Study)

The Trøndelag Health Study (HUNT) is a collaboration between HUNT Research Centre (Faculty of Medicine and Health Sciences, NTNU, Norwegian University of Science and Technology), Trøndelag County Council, Central Norway Regional Health Authority, and the Norwegian Institute of Public Health. The genotyping in HUNT was financed by the National Institutes of Health; University of Michigan; the Research Council of Norway; the Liaison Committee for Education, Research and Innovation in Central Norway; and the Joint Research

Committee between St Olavs hospital and the Faculty of Medicine and Health Sciences, NTNU. Laxmi Bhatta and Ben M. Brumpton received support from the HUNT Center for Molecular and Clinical Epidemiology; Faculty of Medicine and Health Sciences, NTNU; The Liaison Committee for Education, Research and Innovation in Central Norway; and the Joint Research Committee between St Olavs Hospital and the Faculty of Medicine and Health Sciences, NTNU.

10.7. iPSYCH

The iPSYCH consortium is supported by the Lundbeck foundation (grant nos. R1-2=A9118 and R155-2014-1724). The funders had no role in study design, data collection and analysis, decision to publish or preparation of the manuscript.

10.8. LifeLines

The Lifelines Biobank initiative has been made possible by funding from the Dutch Ministry of Health, Welfare and Sport, the Dutch Ministry of Economic Affairs, the University Medical Center Groningen (UMCG the Netherlands), University of Groningen and the Northern Provinces of the Netherlands. The generation and management of GWAS genotype data for the Lifelines Cohort Study is supported by the UMCG Genetics Lifelines Initiative (UGLI). UGLI is partly supported by a Spinoza Grant from NWO, awarded to Cisca Wijmenga. The authors wish to acknowledge the services of the Lifelines Cohort Study, the contributing research centers delivering data to Lifelines, and all the study participants.

The following individuals contributed to the Lifelines study:
Raul Aguirre-Gamboa (1), Patrick Deelen (1), Lude Franke (1), Jan A Kuivenhoven (2), Esteban A Lopera Maya (1), Ilja M Nolte (3), Serena Sanna (1), Harold Snieder (3), Morris A Swertz (1), Peter M. Visscher (3,4), Judith M Vonk (3), Cisca Wijmenga (1)
(1) Department of Genetics, University of Groningen, University Medical Center Groningen, The Netherlands
(2) Department of Pediatrics, University of Groningen, University Medical Center Groningen, The Netherlands
(3) Department of Epidemiology, University of Groningen, University Medical Center Groningen, The Netherlands
(4) Institute for Molecular Bioscience, The University of Queensland, Brisbane, Queensland, Australia.

10.9. MOBA (Norwegian Mother, Father and Child Cohort Study)

The Norwegian Mother, Father and Child Cohort Study (MOBA) is supported by the Norwegian Ministry of Health and Care Services and the Ministry of Education and Research. We are grateful to all the participating families in Norway who take part in this on-going cohort study.

We thank the Norwegian Institute of Public Health (NIPH) for generating high-quality genomic data. This research is part of the HARVEST collaboration, supported by the Research Council

of Norway (#229624). We also thank the NORMENT Centre for providing genotype data, funded by the Research Council of Norway (#223273), South East Norway Health Authorities and Stiftelsen Kristian Gerhard Jebsen. We further thank the Center for Diabetes Research, the University of Bergen for providing genotype data and performing quality control and imputation of the data funded by the ERC AdG project SELECTionPREDISPOSED, Stiftelsen Kristian Gerhard Jebsen, Trond Mohn Foundation, the Research Council of Norway, the Novo Nordisk Foundation, the University of Bergen, and the Western Norway Health Authorities.

This work was supported by in part by Research Council of Norway through its Centre of Excellence funding scheme, grant number 262700.

10.10. NEO (The Netherlands Epidemiology of Obesity Study)

The NEO study is supported by the participating Departments, the Division, and the Board of Directors of the Leiden University Medical Centre, and by the Leiden University, Research Profile Area 'Vascular and Regenerative Medicine'. The authors of the NEO study thank all participants, all participating general practitioners for inviting eligible participants, all research nurses for data collection and the NEO study group: Pat van Beelen, Petra Noordijk and Ingeborg de Jonge for coordination, laboratory and data management.

10.11. NTR (Netherlands Twin Registry)

We warmly thank all twin and family members for their participation.

10.12. QIMR (Queensland Institute of Medical Research)

Data collection funded from various grants from Australian NHMRC and US NIH.

10.13. RS (Rotterdam Study)

The generation and management of GWAS genotype data for the Rotterdam Study (RS I, RS II, RS III) was executed by the Human Genotyping Facility of the Genetic Laboratory of the Department of Internal Medicine, Erasmus MC, Rotterdam, The Netherlands. The GWAS datasets are supported by the Netherlands Organisation of Scientific Research NWO Investments (nr. 175.010.2005.011, 911-03-012), the Genetic Laboratory of the Department of Internal Medicine, Erasmus MC, the Research Institute for Diseases in the Elderly (014-93-015; RIDE2), the Netherlands Genomics Initiative (NGI)/Netherlands Organisation for Scientific Research (NWO) Netherlands Consortium for Healthy Aging (NCHA), project nr. 050-060-810. We thank Pascal Arp, Mila Jhamai, Marijn Verkerk, Lizbeth Herrera and Marjolein Peters, MSc, and Carolina Medina-Gomez, MSc, for their help in creating the GWAS database, and Karol Estrada, PhD, Yurii Aulchenko, PhD, and Carolina Medina-Gomez, MSc, for the creation and analysis of imputed data.

The Rotterdam Study is funded by Erasmus Medical Center and Erasmus University, Rotterdam, Netherlands Organization for the Health Research and Development (ZonMw), the

Research Institute for Diseases in the Elderly (RIDE), the Ministry of Education, Culture and Science, the Ministry for Health, Welfare and Sports, the European Commission (DG XII), and the Municipality of Rotterdam. The authors are grateful to the study participants, the staff from the Rotterdam Study and the participating general practitioners and pharmacists.

10.14. SHIP (Study of Health in Pomerania)

SHIP is part of the Community Medicine Research net of the University of Greifswald, Germany, which is funded by the Federal Ministry of Education and Research (grants no. 01ZZ9603, 01ZZ0103, and 01ZZ0403), the Ministry of Cultural Affairs as well as the Social Ministry of the Federal State of Mecklenburg-West Pomerania, and the network 'Greifswald Approach to Individualized Medicine (GANI_MED)' funded by the Federal Ministry of Education and Research (grant 03IS2061A). Genome-wide data have been supported by the Federal Ministry of Education and Research (grant no. 03ZIK012) and a joint grant from Siemens Healthineers, Erlangen, Germany and the Federal State of Mecklenburg- West Pomerania. The University of Greifswald is a member of the Caché Campus program of the InterSystems GmbH.

10.15. STR (Swedish Twin Registry)

The Swedish Twin Registry is managed by Karolinska Institutet and receives funding through the Swedish Research Council under the grant no 2017-00641. Genotyping was performed by the SNP&SEQ Technology Platform in Uppsala (www.genotyping.se). The facility is part of the National Genomics Infrastructure supported by the Swedish Research Council for Infrastructures and Science for Life Laboratory, Sweden. The SNP&SEQ Technology Platform is also supported by the Knut and Alice Wallenberg Foundation.

10.16. UKHLS (Understanding Society)

University of Essex, Institute for Social and Economic Research. (2022). Understanding Society: Waves 1-12, 2009-2021 and Harmonised BHPS: Waves 1-18, 1991-2009. [data collection]. 17th Edition. UK Data Service. SN: 6614, http://doi.org/10.5255/UKDA-SN-6614-18.

11. References

1. Kweon, H. *et al.* Genetic Fortune: Winning or Losing Education, Income, and Health. *TI Discuss. Pap. Ser.* **20**, (2020).
2. The 1000 Genomes Project Consortium. An integrated map of genetic variation from 1,092 human genomes. *Nature* **491**, 56–65 (2012).
3. McCarthy, S. *et al.* A reference panel of 64,976 haplotypes for genotype imputation. *Nat. Genet.* **48**, 1279 (2016).
4. Loh, P.-R. *et al.* Efficient Bayesian mixed-model analysis increases association power in large cohorts. *Nat. Genet.* **47**, 284–290 (2015).
5. Jiang, L. *et al.* A resource-efficient tool for mixed model association analysis of large-scale data. *Nat. Genet.* **51**, 1749–1755 (2019).
6. Winkler, T. W. *et al.* Quality control and conduct of genome-wide association meta-analyses. *Nat. Protoc.* **9**, 1192–1212 (2014).
7. Lee, J. J. *et al.* Gene discovery and polygenic prediction from a genome-wide association study of educational attainment in 1.1 million individuals. *Nat. Genet.* **50**, 1112 (2018).
8. Okbay, A. *et al.* Genome-wide association study identifies 74 loci associated with educational attainment. *Nature* **533**, 539–542 (2016).
9. Karlsson Linnér, R. *et al.* Genome-wide association analyses of risk tolerance and risky behaviors in over 1 million individuals identify hundreds of loci and shared genetic influences. *Nat. Genet.* **51**, 245–257 (2019).
10. Bulik-Sullivan, B. *et al.* An atlas of genetic correlations across human diseases and traits. *Nat. Genet.* **47**, 1236–1241 (2015).
11. Grotzinger, A. D. *et al.* Genomic structural equation modelling provides insights into the multivariate genetic architecture of complex traits. *Nat. Hum. Behav.* **3**, 513–525 (2019).
12. The International HapMap 3 Consortium. Integrating common and rare genetic variation in diverse human populations. *Nature* **467**, 52 (2010).
13. Watanabe, K., Taskesen, E., Van Bochoven, A. & Posthuma, D. Functional mapping and annotation of genetic associations with FUMA. *Nat. Commun.* **8**, 1–11 (2017).
14. Okbay, A. *et al.* Genetic variants associated with subjective well-being, depressive symptoms, and neuroticism identified through genome-wide analyses. *Nat. Genet.* **48**, 624–633 (2016).
15. Frei, O. *et al.* Bivariate causal mixture model quantifies polygenic overlap between complex traits beyond genetic correlation. *Nat. Commun.* **10**, 2417 (2019).
16. Demange, P. A. *et al.* Investigating the genetic architecture of noncognitive skills using GWAS-by-subtraction. *Nat. Genet.* **53**, 35–44 (2021).
17. Okbay, A. *et al.* Polygenic prediction of educational attainment within and between families from genome-wide association analyses in 3 million individuals. *Nat. Genet.* 1–13 (2022) doi:10.1038/s41588-022-01016-z.
18. Hill, W. D. *et al.* Molecular Genetic Contributions to Social Deprivation and Household Income in UK Biobank. *Curr. Biol.* **26**, 3083–3089 (2016).
19. Hill, W. D. *et al.* Genome-wide analysis identifies molecular systems and 149 genetic loci associated with income. *Nat. Commun.* **10**, 1–16 (2019).
20. Yang, J., Lee, S. H., Goddard, M. E. & Visscher, P. M. GCTA: A Tool for Genome-wide Complex Trait Analysis. *Am. J. Hum. Genet.* **88**, 76–82 (2011).
21. Privé, F., Arbel, J. & Vilhjálmsson, B. J. LDpred2: better, faster, stronger. *Bioinformatics* **36**, 5424–5431 (2020).
22. Vilhjálmsson, B. J. *et al.* Modeling Linkage Disequilibrium Increases Accuracy of

Polygenic Risk Scores. *Am. J. Hum. Genet.* **97**, 576–592 (2015).
23. Kong, A. *et al.* The nature of nurture: Effects of parental genotypes. *Science* **359**, 424–428 (2018).
24. Trejo, S. & Domingue, B. W. Genetic nature or genetic nurture? Introducing social genetic parameters to quantify bias in polygenic score analyses. *Biodemography Soc. Biol.* **64**, 187–215 (2018).
25. Morris, T. T., Davies, N. M., Hemani, G. & Smith, G. D. Population phenomena inflate genetic associations of complex social traits. *Sci. Adv.* **6**, eaay0328 (2020).
26. Young, A. I. *et al.* Mendelian imputation of parental genotypes improves estimates of direct genetic effects. *Nat. Genet.* **54**, 897–905 (2022).
27. Manichaikul, A. *et al.* Robust relationship inference in genome-wide association studies. *Bioinformatics* **26**, 2867–2873 (2010).
28. Loh, P.-R. *et al.* Reference-based phasing using the Haplotype Reference Consortium panel. *Nat. Genet.* **48**, 1443–1448 (2016).
29. Timmers, P. R. *et al.* Genomics of 1 million parent lifespans implicates novel pathways and common diseases and distinguishes survival chances. *elife* **8**, e39856 (2019).
30. Jones, S. E. *et al.* Genome-wide association analyses of chronotype in 697,828 individuals provides insights into circadian rhythms. *Nat. Commun.* **10**, 343 (2019).
31. Liu, M. *et al.* Association studies of up to 1.2 million individuals yield new insights into the genetic etiology of tobacco and alcohol use. *Nat. Genet.* **51**, 237–244 (2019).
32. Walters, R. K. *et al.* Transancestral GWAS of alcohol dependence reveals common genetic underpinnings with psychiatric disorders. *Nat. Neurosci.* **21**, 1656–1669 (2018).
33. Yengo, L. *et al.* Meta-analysis of genome-wide association studies for height and body mass index in~ 700000 individuals of European ancestry. *Hum. Mol. Genet.* **27**, 3641–3649 (2018).
34. Xue, A. *et al.* Genome-wide association analyses identify 143 risk variants and putative regulatory mechanisms for type 2 diabetes. *Nat. Commun.* **9**, 2941 (2018).
35. Graham, S. E. *et al.* The power of genetic diversity in genome-wide association studies of lipids. *Nature* **600**, 675–679 (2021).
36. Demontis, D. *et al.* Discovery of the first genome-wide significant risk loci for attention deficit/hyperactivity disorder. *Nat. Genet.* **51**, 63–75 (2019).
37. Mullins, N. *et al.* Genome-wide association study of more than 40,000 bipolar disorder cases provides new insights into the underlying biology. *Nat. Genet.* **53**, 817–829 (2021).
38. Ruderfer, D. M. *et al.* Genomic dissection of bipolar disorder and schizophrenia, including 28 subphenotypes. *Cell* **173**, 1705–1715 (2018).
39. Grove, J. *et al.* Identification of common genetic risk variants for autism spectrum disorder. *Nat. Genet.* **51**, 431–444 (2019).
40. Watson, H. J. *et al.* Genome-wide association study identifies eight risk loci and implicates metabo-psychiatric origins for anorexia nervosa. *Nat. Genet.* **51**, 1207–1214 (2019).
41. Arnold, P. D. *et al.* Revealing the complex genetic architecture of obsessive-compulsive disorder using meta-analysis. *Mol. Psychiatry* **23**, 1181–1181 (2018).
42. Howard, D. M. *et al.* Genome-wide meta-analysis of depression identifies 102 independent variants and highlights the importance of the prefrontal brain regions. *Nat. Neurosci.* **22**, 343–352 (2019).
43. Purves, K. L. *et al.* A major role for common genetic variation in anxiety disorders. *Mol. Psychiatry* **25**, 3292–3303 (2020).

44. Nagel, M. *et al.* Meta-analysis of genome-wide association studies for neuroticism in 449,484 individuals identifies novel genetic loci and pathways. *Nat. Genet.* **50**, 920–927 (2018).
45. Meier, S. M. *et al.* Genetic variants associated with anxiety and stress-related disorders: a genome-wide association study and mouse-model study. *JAMA Psychiatry* **76**, 924–932 (2019).
46. Johnson, E. C. *et al.* A large-scale genome-wide association study meta-analysis of cannabis use disorder. *Lancet Psychiatry* **7**, 1032–1045 (2020).
47. Lee, P. H. *et al.* Genomic Relationships, Novel Loci, and Pleiotropic Mechanisms across Eight Psychiatric Disorders. *Cell* **179**, 1469-1482.e11 (2019).
48. The GTEx Consortium *et al.* The Genotype-Tissue Expression (GTEx) pilot analysis: Multitissue gene regulation in humans. *Science* **348**, 648–660 (2015).
49. Ramasamy, A. *et al.* Genetic variability in the regulation of gene expression in ten regions of the human brain. *Nat. Neurosci.* **17**, 1418–1428 (2014).
50. Finucane, H. K. *et al.* Heritability enrichment of specifically expressed genes identifies disease-relevant tissues and cell types. *Nat. Genet.* **50**, 621–629 (2018).
51. Leeuw, C. A. de, Mooij, J. M., Heskes, T. & Posthuma, D. MAGMA: Generalized Gene-Set Analysis of GWAS Data. *PLOS Comput. Biol.* **11**, e1004219 (2015).
52. Fehrmann, R. S. N. *et al.* Gene expression analysis identifies global gene dosage sensitivity in cancer. *Nat. Genet.* **47**, 115–125 (2015).
53. Wei, W.-Q. *et al.* Evaluating phecodes, clinical classification software, and ICD-9-CM codes for phenome-wide association studies in the electronic health record. *PloS One* **12**, e0175508 (2017).
54. Wu, P. *et al.* Mapping ICD-10 and ICD-10-CM Codes to Phecodes: Workflow Development and Initial Evaluation. *JMIR Med. Inform.* **7**, e14325 (2019).

1. Frequently Asked Questions (FAQs)

This document provides information about the study:

Kweon *et al.* (2024) "Associations between common genetic variants and income provide insights about the socio-economic health gradient."

This FAQ was prepared by several of the study's coauthors and draws from and builds on the FAQs of SSGAC papers, as well as the FAQ of Demange et al. 2020[1], which was written by one of the co-authors of the present study, Paige Harden. Questions and comments about the paper or this FAQ should be sent to Philipp Koellinger (p.d.koellinger@vu.nl) or Abdel Abdellaoui (a.abdellaoui@amsterdamumc.nl).

2. Summary

- We investigated associations between genetics and income and explored how these are linked to health outcomes. By analyzing data from 668,288 individuals in 12 affluent countries who carried genotypes most similar to the EUR reference panel of the 1000 Genomes dataset (1KG-EUR), we discovered that different measures of income share common genetic associations. These associations do not imply a direct genetic determination of income, but probably reflect the ways in which a particular society may reward certain genetic predispositions. We identified 162 genetic regions associated with these income measures, 88 of which were previously unknown to be linked to income. The effect of each individual genetic region is very small.
- We observed substantial heterogeneity in the genetic architecture of income across cohorts and non-perfect genetic correlations of income across sexes. This underlines that the genetic associations we report here are averages across different groups and environments that should not be interpreted as fixed or universal.
- By creating a polygenic index based on our genetic association results, we were able to capture 1 - 4% of the variation in income measures among 1KG-EUR-like individuals. The predictive power of the polygenic index decreased by ~75% when accounting for the polygenetic indexes of an individual's parents, indicating that assortative mating magnifies the predictive power of the polygenic index relative to the causal effects of the genetic variants and/or that environmental factors correlated with the family account for a substantial part of the link between genetic variants and income. The predictive power of the PGI is substantially reduced in non-EUR samples, showing that our results have limited generalizability to different ancestry groups.
- Our analysis revealed that associations between higher income and better health outcomes are partly due to common genetic factors linked to both. Genetic effects associated with higher income correlate with lower BMI, blood pressure, type-2 diabetes, depression, and reduced stress-related disorders. Interestingly, genetic components of income not shared with educational attainment are related to better mental health, but reduced physical health benefits and increased risky behaviours such as drinking and smoking.
- We found genetic correlations between a range of psychiatric disorders and both the overall genetic effects associated with income and those specific to income - those not overlapping with educational attainment. Notably, for certain disorders - schizophrenia, autism, and obsessive-compulsive disorder - these correlations were <u>positive</u> when considering all genetic effects associated with income and educational attainment, and <u>negative</u> for those unique to income. This may indicate that the educational system may better accommodate individuals with these disorders than the labor market does, or that talents associated with these genetic risks are advantageous in school but not in the labor market.

- It is of paramount importance to stress that our study does not provide any basis for asserting inherent superiority or accepting social inequality as an inevitability grounded in genetics. We firmly reject such notions on scientific and ethical grounds. Instead, our findings demonstrate that genetic endowments play a role in inequality and that their effects are influenced by environmental and societal factors that are susceptible to change. Moreover, our study results should not be used for making comparisons between different groups or predicting individual-level outcomes. Discrimination cannot be justified based on genetic associations.
- In summary, our study sheds light on the complex interplay between genetics, socio-economic status, and health outcomes. It underscores the importance of environmental and societal factors in these relationships, and supports the imperative for fairness in access to opportunities and resources for all individuals.

3. The current study

3.1. What is the purpose of this study?

Understanding the causes of social mobility and in particular the structural sources of inequality is of fundamental importance both as a matter of science and of social policy.[2] Poverty and economic deprivation are major risk factors for mental and physical diseases,[3] lower life expectancy,[4] and lower well-being.[5] Furthermore, socio-economic status (SES) is important to health not only for those in poverty, but at all levels of SES.[6] It has long been recognized that parental SES is a major determinant of a child's expected trajectory in terms of cognitive and non-cognitive skill development, behaviors,[7] educational attainment,[8] career prospects, and adult income.[9] In other words, differences in SES are partially transmitted across generations. At the same time, education, income, personality, cognitive abilities, and occupational choices are all heritable to some extent and parents pass on both their environments and their genes to their offspring.[10–13] Consequently, disentangling the effects of behavior, environment, and genetics on income poses a substantial scientific challenge, yet remains important for comprehending social mobility and gaining insights into the intricate relationships between income and health.

Yet, the scientific possibilities to do so are limited. In the past, the primary tools to disentangle the effects of a parent's genes from parental environment were adoption studies[14] and children-of-twin studies.[15] However, few samples of this type exist, those that do are typically small, and these naturally occurring experiments are rarely representative of the entire range of environments. Furthermore, these datasets do not allow the investigation of any interactions between environments and specific biological pathways. As a result, scientific insights are still very limited about why and how social inequalities tend to persist within families throughout generations, why and how these inequalities translate into differences in health and mortality, and what the most effective ways are to help disadvantaged individuals.

With the advent of well-powered genome-wide association studies (GWAS) on socio-economic indicators such as educational attainment and income, new opportunities have emerged to help address these challenges. Polygenic indices derived from GWAS offer new approaches to investigate the contributions of direct genetic effects and environmentally mediated mechanisms in intergenerational social mobility, for example in samples of trios comprising mother, father, and child.[16] Additionally, well-powered GWAS summary statistics for a wide range of traits enable the exploration of genetic effects shared between income and health outcomes, with the potential to unveil previously unknown relationships. They also facilitate studies investigating interaction effects between genetic and

environmental factors. Furthermore, incorporating polygenic indices from robust GWAS on income can help control for potential genetic confounds and enhance the statistical power of social scientific studies on income and social mobility.[17,18]

The goal of our study was to provide additional insights into common genetic variants associated with income, to shed light on potential societal biases towards certain genetic predispositions, and to gain information about the intricate relationships between health and socio-economic status. In particular, we used advanced statistical methods to disentangle the genetic associations with income and educational attainment (EA) and leveraged them to gain insights into distinct associations of these two major indicators of SES with health outcomes. We are sharing the GWAS summary statistics of our study with the broader research community. With this new data, researchers will be able to study a variety of important questions that will be informative about the structural sources of inequality and their relationships with health.

3.2. What did you do in this paper?

We conducted a GWAS (What is a GWAS?) of income in a sample of over 600,000 participants. To construct such a large sample, we collected 32 datasets from 12 countries. All of these datasets have surveyed and genotyped their research participants. We considered four income measures (individual, household, occupational, parental). We conducted a GWAS for each income measure and combined the results.

We then used the findings of our GWAS to perform additional analyses that explored:

1. the environmental heterogeneity in the genetic factor for income, by comparing the results by sex and by countries,
2. similarities and differences of our results with the most recent GWAS for educational attainment,
3. the predictive power of the polygenic index for income as well as the influence of family environment, and
4. the relation with other behavioral and health-related phenotypes.

3.3. How was income measured in the current study?

Our GWAS used four measures of income: individual, occupational, household, and parental income.

Individual income is the most direct measure of the consumption and savings opportunities that a person has. Individual income is the result of various factors including achieved qualifications (e.g., education, learned occupation, experience), personal characteristics (e.g., leadership, cognitive skills, consciousness), the demand and supply for these qualifications and characteristics in the labor market, and personal choices about labor supply (e.g., due to personal preferences, and decisions about division of labor among household members).

However, most large datasets that contain genetic information do not have measures of individual income. To address this challenge, we used three additional measures of income (household income, parental income, occupational income) that are all genetically highly correlated (**Fig. 1**). Household income was measured by questionnaires. Parental income (only available in the Danish iPsych sample) was derived from national registries, calculating the average income of mothers and fathers between ages 30 and 55. Occupational income was derived from standardized occupational codes of participants. Using the national statistics based on the same occupational coding, we imputed the average income within occupational codes for each participant.

3.4. Did you find the gene(s) for income?

No.

We did not find "the gene for" income. We identified many genetic variants that are associated with income. Although it was once believed that scientists would discover numerous one-to-one associations between genes and outcomes, we have known for many years that the vast majority of human traits and other outcomes are complex and are influenced by many (thousands or even tens of thousands of) genes, each of which alone tends to have a small influence on the relevant outcome.[20]

Although we did find several genes that are associated with income, we believe that characterizing these as "genes for income" is likely to mislead, for many reasons.

First, a large part of the variation in people's income is accounted for by social and other environmental factors, not by additive genetic effects. "Genes for income" might be read to imply, incorrectly, that genes are the strongest predictor of variation in income — this is not the case.

Second, the genetic variants that are associated with income are also associated with many other things (only some of which we identify in this study, see, for example, our

results on the links between income and health). These variants are no more "for" income than for the other outcomes with which they are associated (e.g., educational attainment, BMI, Alzheimer's disease, HDL cholesterol levels).

Third, each individual genetic variant captures only a tiny part of the variance in income (less than 0.011%). Our results suggest that hundreds, or even thousands, of genetic variants are associated with income, but each of them considered by itself has only a tiny effect. The phrase "genes for income" might misleadingly imply large effects of specific genes, but these effects do not exist.

Fourth, environmental factors can increase or decrease the impact of specific genetic variants. Put differently, even if a genetic variant is associated with higher or lower levels of income *on average*, it may have a much larger or smaller effect depending on social and environmental conditions. We illustrate this by calculating the *average* genetic correlation of income across the various datasets included in our meta-analysis. For individual income, this average genetic correlation across samples is only 0.45, suggesting a high degree of heterogeneity in the genetic architecture of income across different environments.

Finally, genes do not affect income directly. Rather, their influence works via social and environmental channels that are subject to change. For example, our results suggest that the associations between genes and income work partly through educational attainment which can be influenced by policy interventions. Furthermore, the predictive power of the polygenic index decreased by ~75% when accounting for the genetic indexes of an individual's parents, indicating that family environments account for a substantial part of the link between genetic variants and income.

3.5. Are the genetic variants associated with income in your study also associated with other outcomes?

Yes.

3.6. How good is your polygenic index?

Our polygenic index captures approximately 1 - 4% of the variance in different income measures in three hold-out samples from the United Kingdom, the United States, and Sweden (see also the FAQ section What is a polygenic index?). This is good enough for important research purposes such as exploring the relationships between health and income

but is useless for (misguided) attempts to "predict" individual-level outcomes from genetic data (see section 3.2 below). The predictive accuracy of our polygenic index is substantially lower in non-EUR samples, illustrating the limited generalizability of our results to non-European ancestry groups.

Note that our estimates of the "SNP heritability" of income (Supplementary Table 13) suggest a cohort-specific upper bound of 10~13% for the potential accuracy of polygenic indices for income in the future, as GWAS sample becomes larger (see the FAQ What is a GWAS?). Even a polygenic index of ~10% would not be accurate enough to make meaningful statistical predictions at the individual level — see Figure 3 in [18]. Furthermore, our prediction analyses suggest that approximately three-fourths of the signal our polygenic index picks up is actually due to environmental effects that are correlated with genes, such as the rearing environment provided by one's parents. Even the remaining effects are partially mediated by environmental channels such as educational attainment. This finding clearly illustrates what we said here earlier — polygenic indices are not a clean way to separate biological and environmental influences. **Genetic effects on socioeconomic outcomes such as income do not exist in a vacuum — they are shaped by environmental conditions that keep evolving and that are not equal for everyone.**

3.7. What does your study *not* mean?

Genetic research has a long history of being misinterpreted and misused to argue that social inequality is inevitable, that social programs designed to improve people's lives are bound to fail and that some people are "naturally" inferior to other people.[22] **We wholeheartedly reject these claims on both scientific and moral grounds.**

A high or low polygenic index should not be interpreted to mean that someone is destined or determined to show a particular characteristic. It is not a "fortune teller", nor a pure measure of someone's genetic "endowment." It is just one of many factors that matter. By way of analogy, having high cholesterol makes it more likely that an individual will have a heart attack, but it does not determine that outcome — lots of people have high cholesterol but don't have a heart attack, and you can take steps to prevent a heart attack if you are at high risk. Similarly, a high polygenic index means that an individual has a slightly higher probability of obtaining a higher income, but that higher probability does not mean destiny.

Genetic associations with income do not mean that the environment does not make a difference. In fact, polygenic indices such as ours partly capture what most people would consider as environmental effects. If, for example, parents with particular genetic variants

are more likely to live in a wealthy neighborhood with good schools, and if good schools make it more likely that children will get good jobs later in their lives, then this means that their children's polygenic indices for income will be correlated with the quality of schools they attended. Our analyses show that roughly three-fourths of the signal our polygenic index for income picks up is due to such indirect genetic effects or other environmental influences that just happen to be correlated with genes.

Genetic associations with income do not mean that interventions or policy reforms designed to combat inequalities are bound to fail. For example, interventions such as the Perry Preschool Project have shown that access to high-quality education is an effective way to improve lifetime outcomes, including a higher likelihood of getting a good job that pays well.

The existence of genetic associations with income within a group of people does not tell us anything about whether there are average differences between racial or ethnic groups, or why such differences, if they are observed, occur. This is an important point, because racist and classist ideas about the allegedly "inferior" character of people of color and the poor have been used to justify eugenic policies.[23–25] Nothing about this study gives any sort of empirical support to these ideas.

3.8. Is this the first GWAS on income?

No.

Two previous GWAS have looked at household income in the UK Biobank. These studies uncovered many interesting findings that help to illuminate the complex relationships between socioeconomic conditions and health.[26,27] The current study is based on a substantially larger sample size that we obtained by pooling data from 32 different samples across 12 economically advanced countries and three continents. The larger sample size allows us to discover additional genetic variants associated with income, yields a better-performing polygenic index, and allows more precise estimates of the genetic correlations of income with other traits. We also used a multivariate statistical approach that helps us to parse genetic associations with income that are also related with educational attainment and those that are not. We used the results of the latter analyses to gain novel insights into partly divergent health-implications of educational attainment and income.

4. Implications of the study

4.1. Does this study show that an individual's level of income is determined, or fixed, at conception?

No.

Social and other environmental factors are the main drivers of variation in income. However, even if it were true that genetic factors accounted for *all* of the differences among individuals in income, it would *still* not follow that an individual's income is "determined" at conception. There are at least three reasons for this:

First, some (if not all) genetic effects may operate through environmental channels.[28] Our study clearly illustrates the relevance of educational attainment for income, and education can be changed through environmental interventions (e.g., policy).

Second, even if the genetic associations with income operated entirely through non-environmental mechanisms that are difficult to modify (such as direct influences on the formation of neurons in the brain and the biochemical interactions among them), powerful environmental interventions could still change these genetic relationships. In a famous example suggested by the economist Arthur Goldberger, even if all variation in unaided eyesight were due to genes, there would still be enormous benefits from introducing eyeglasses.[29] Similarly, policies that guarantee a minimum wage or a basic income have incontrovertible relevance for living standards and the overall distribution of income.

4.2. Can your polygenic index be used to predict how well someone will do in life?

No (see also the FAQ sections What is a polygenic index? and How good are your polygenic indices?).

The figure below illustrates the statistical reason why the polygenic index cannot be used to predict how well someone will do in life. It shows data from the Health and Retirement Study, one of our replication samples in our paper. The *x*-axis plots the values of the Income Factor PGI among 6,171 individuals. The values of the index in that sample were standardized to have a mean of zero and a standard deviation of one. The *y*-axis plots values of log self-reported income, controlling for demographic variables (see the figure note). Each dot in the figure represents the combination of the polygenic index and income for one specific person in the sample. Despite the small, positive relationship between the index and income in the sample (indicated by the dotted red regression line), it can be seen

clearly that the values of the index are not very informative about the income of any specific individual in the sample. In fact, a very broad range of income values is observed even for people who have very low or very high values of the polygenic index (e.g., those that are two standard deviations away from the mean).

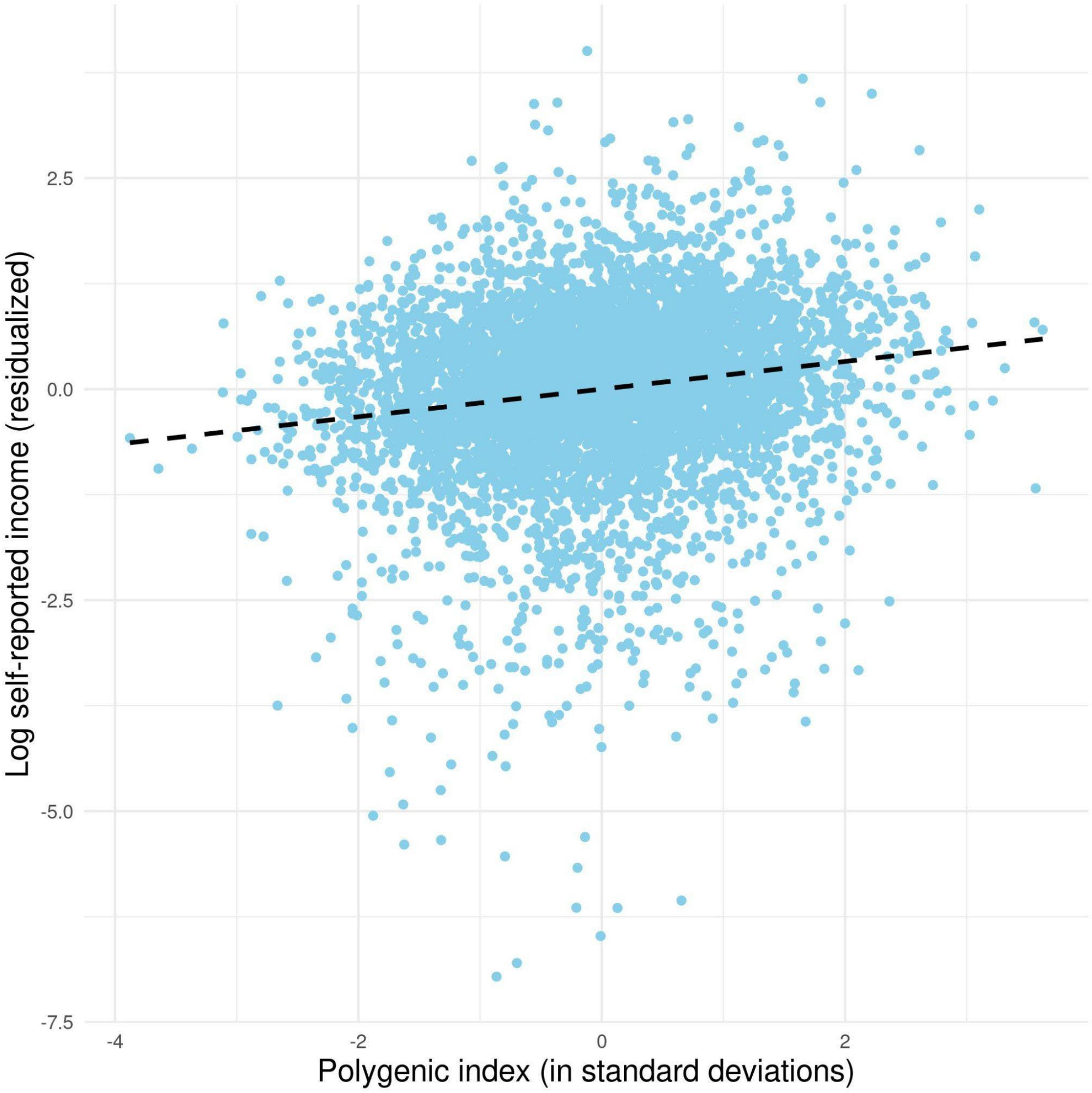

Note: The figure presents a scatter plot for the Health and Retirement Study (*N*=6,171). The x-axis shows standardized values of the Income Factor PGI, constructed from a GWAS meta-analyses that excluded the Health and Retirement Study. The y-axis shows log self-reported income, which was constructed as follows: within each wave, the log income was regressed on demographic variables (sex, age, age^2, age^3, and the interactions between sex and the age terms) and genetic control variables (top 20 genetic principal components and genotyping batches). Then, the mean of the residuals from the regressions was obtained for each individual, which was then standardized. The dotted line is a regression line with slope 0.172 ($p = 5.3 \times 10^{-42}$).

4.3. Can your polygenic index be used for research studies in non-European-ancestry populations?

Only in a very limited way (see Supplementary Fig. 3 and Supplementary Information section 5.3).

As a practical matter, it is possible to calculate a polygenic index for any individual for whom genome-wide data are available, but the polygenic index is expected to be much less accurate for non-European-ancestry populations.[30] For example, a polygenic index for educational attainment in a previous study constructed from a GWAS in over 3 million 1KG-EUR-liked individuals captures 12.0% of the variance in years of schooling among individuals with similar ancestries in the US Health and Retirement Study. The same index captures only 1.3% of the variance in years of schooling among African-Americans in the same sample.[31] For income, we observed reduction in predictive accuracy from 4-5% among European ancestry samples to 0-2% among Africans, Caribbeans, Indians, East Asians, and South Asians (see Supplementary Fig. 3 and Supplementary Information section 5.3).

Our study was conducted only using 1KG-EUR-like samples of individuals (see the FAQ section Quality control measures). This choice was based primarily on data availability since the vast majority of all genotyped samples at the time of our study are 1KG-EUR-like (colloquially referred to as "European ancestry" below).[39] The set of SNPs that are associated with income in individuals of European ancestry is unlikely to overlap perfectly with the set of SNPs associated with income sampled in other parts of the world. Even if a given SNP is associated in both ancestry groups, the effect size—in other words, the strength of the association—will almost surely differ, partly because linkage disequilibrium (LD) patterns (i.e., the correlational structure of the genome) vary by ancestry.[32] Thus, some variants may be associated with income because the variant is in LD (i.e., correlated) with a variant elsewhere in the genome that causally affects income (What is a GWAS? Are the genetic variants identified in a GWAS "causal"?). If the strength of the correlation is greater in one ancestry group than in another, then the size of the association will be larger in that ancestry group. Moreover, even if LD patterns were similar in each ancestry group, the association may differ in different groups because the social and environmental conditions differ (What is a polygenic index?). Different socio-economic systems may assign differing values to certain genetic predispositions, impacting income potential. Thus, a genetic predisposition that increases income generation in one group or society may not have the same effect in another. The fact that there are differences across ancestry groups in the set of associated SNPs and their effect sizes has two important implications.

First, it means that the *polygenic indices of individuals from different ancestry groups cannot be meaningfully compared*. A recent paper[30] illustrated this point in the context of polygenic indices for predicting height; in the sample analyzed in that paper, polygenic

indices for height for individuals of European ancestry are on average larger than those of South Asian ancestry which in turn are larger than those of African ancestry. In actuality, however, populations of African ancestries represented by the sample have similar heights to populations of European ancestries, and both African and European populations tend to be taller than South Asian populations.

Second, while polygenic indices may be used to study differences across individuals within a sample of people of non-European-ancestries, the accuracy of the index will be much smaller than that in a sample of people of European ancestries. Such an attenuation of predictive power has been repeatedly found in prior work.[33–36]

Third, the existence of genetic associations with income within a group of people (in our study, individuals of European ancestries) does not tell us anything about whether there are average differences between racial or ethnic groups, or why such differences, if they are observed, occur. This is an important point, because racist and classist ideas about the allegedly "inferior" character of people of color and the poor have been used to justify eugenic policies.[23–25] Nothing about this study gives any sort of empirical support to these ideas.

For a more extensive, excellent discussion of these and related issues, see Graham Coop's blog post "Polygenic scores and tea drinking": https://gcbias.org/2018/03/14/polygenic-scores-and-tea-drinking/.

4.4. What policy lessons do you draw from this study? How could society benefit from this work?

We do not make direct recommendations for policy in this paper.

However, our results show that how well people do in life depends to some degree the "genetic lottery"[38] (i.e., which particular combination of your parents' genes you inherited). No one decides to participate in these lotteries or has any influence on its outcome. Our within-family analyses demonstrate that random genetic differences between siblings influence how they earn and how healthy they are (**Extended Fig. 5**). Although the overall effect sizes of these random genetic difference on income and health are small, the existence of them limits the extent to which people can take personal credit for the good things that happen to them and may inspire some humility. It also puts limits on how much people can be blamed for things that did not turn out in their favor. Furthermore, our results show that luck and agency (e.g., the choices people make, and how hard they work) cannot be clearly separated from one another — our polygenic index is associated with education and income, two measures that many may intuitively file under effort rather than luck. Our hope is that the insight that (genetic and social) luck matters may contribute to a greater sense of solidarity in society, more empathy for those who are unlucky, and a greater

willingness to share among those who have the means to give. This is our preferred interpretation of our empirical results, which is in line with arguments put forward in moral philosophy[39,40] and with empirical studies that found that people are less willing to tolerate inequality that is due to luck rather than effort.[41–46] However, this is not the only way to interpret our results and different policy implications may be derived from different philosophical perspectives.[47]

In general, the widespread availability of genetic data and the increasing insights into how genetic factors are associated with observed differences between people creates new challenges for policy making across various domains, including insurance markets, labor markets, personalized medicine, and reproductive technologies. In many of these areas, societies and policy makers will have to make difficult and complex trade-offs involving the protection of human rights, including respect for autonomy, dignity, privacy, the right to science, the right to know or not to know about genetic results, as well as the feasibility of certain forms of insurance or possible improvements in health and well-being.[48] One of the authors of the current study, Koellinger, recently wrote a policy report for the European Commission that reviews some of these challenges for policy making. This report can be accessed here.

We would like to state explicitly and emphatically that the genetic association results reported in the current study should not be used for any form of individual selection or discrimination (e.g., in labor or insurance markets or in reproductive technologies); see the FAQ sections What does your study not mean?, Can your polygenic index be used to predict how well someone will do in life?, and Can your polygenic index be used for research studies in non-European-ancestry populations? .

4.5. Could this kind of research lead to discrimination against, or stigmatization of, people with the relevant genetic variants? If so, why conduct this research?

Unfortunately, like a great deal of research—including, for instance, research identifying genomic variants associated with increased cancer risk—the results can be misunderstood and misapplied. This includes misuses that would discriminate against people who carry specific genetic variants (e.g., in insurance or labor markets). Nevertheless, for a variety of reasons, we do not believe that the best response to the possibility that useful knowledge might be misused is to refrain from producing that knowledge. Furthermore, we do not claim that genetically-informed studies are better than other empirical approaches. However, we briefly discuss some of the broad potential benefits of this research. We then describe what we take to be our ethical obligation as researchers conducting this work.

First, a benefit of conducting social science genetics research in ever larger samples is that doing so allows us to correct the scientific record. An important theme in earlier work by the SSGAC has been to point out that most existing studies in social-science genetics

that report genetic associations with behavioral traits have serious methodological limitations, fail to replicate and are likely to be false-positive findings.[49–52] This same point was made in an editorial in *Behavior Genetics* (the leading journal for the genetics of behavioral traits), which stated that "it now seems likely that many of the published [behavior genetics] findings of the last decade are wrong or misleading and have not contributed to real advances in knowledge".[53] One of the most important reasons why earlier work has generated unreliable results is that the sample sizes were far too small, given that the true effects of individual genetic variants on behavioral traits are small.

Second, social science genetics also has the potential to correct the *social* record and thereby to help *combat* discrimination and stigmatization. Our study clearly demonstrates that (i) the vast majority of income inequality is due to environmental factors, and (ii) the genetic influences we identified are not deterministic. Instead, they also depend on environmental factors and work via environmental channels that can be influenced (e.g., via educational attainment).

Third, social science genetics research has the potential to yield many other benefits, as briefly summarized in the FAQ section Where can I learn more about social science genetics? and further explained in [18]. Foregoing this research necessarily entails foregoing these and any other possible benefits, some of which will likely be the result of serendipity rather than being foreseeable.

In sum, we agree with the U.K. Nuffield Council on Bioethics, which concluded in a report [54], p.114) that "research in behavioural genetics has the potential to advance our understanding of human behaviour and that the research can therefore be justified," but that "researchers and those who report research have a duty to communicate findings in a responsible manner." In our view, responsible behavioral genetics research includes a sound methodology and analysis of data; a commitment to publish all results, including any negative results; and the transparent, complete reporting of the methodology and findings in publications, presentations, and communications with the media and the public, including particular vigilance regarding what the results do—and do not—show (hence, this FAQ document).

5. Background

5.1. What does it mean to say that income is "heritable"?

"Heritable" is a confusing word that is often misinterpreted to mean that heritable characteristics are determined by biology. This is not true. As we describe in our article in depth, heritable characteristics — including income — *can* be influenced by the environment, *can* be changed by interventions or policy reforms, and are *not* "destined" to develop in a particular way based just on a person's genes. "Heritable" means that, within the sample of people being studied, those who are more genetically different from each other tend to show more different characteristics.[55] We return to this point — that heritable characteristics are not biologically determined — in the FAQ sections What is a GWAS? Are the genetic variants identified in a GWAS "causal"? and What does your study not mean?.

Previous studies have measured income and similar traits in identical and fraternal twins, and found that identical twins are more similar in their income skills than fraternal twins are.[56] This result tells us that income has a heritable component. Using different data and a different approach (i.e., by quantifying whether observed genetic differences between individuals correspond to differences in income), we come to the same conclusion in our study. Because we now know that virtually all aspects of behavior and personality that differ between people are at least partly heritable, this result is not surprising.[12,57] The heritability of income should not be taken to mean that income is especially "genetic" in some way.

5.2. What is a GWAS? Are the genetic variants identified in a GWAS "causal"?

The short answers to these questions are as follows, with links to more details listed below:

- GWAS (genome-wide association studies) systematically scan the entire human genome for associations with a trait of interest, one SNP at a time.
- Correlations between SNPs are ignored.
- Not all potentially relevant genetic variants are included in a GWAS, partly because they may not have been measured precisely enough or measured at all.
- How frequently a particular genetic variant occurs in a population varies across places, which implies that genes and environments are correlated.

- GWAS try to control for potential correlations between genes and environments, but most GWAS (including ours) do so only imperfectly.
- Genes often influence outcomes only indirectly via environmental and behavioral pathways that can be changed.
- **For all of the reasons above, genetic variants identified in a GWAS are generally not directly causal**.

In a genome-wide association study (GWAS), scientists look at genetic variants measured across the entire human genome to see whether any of them are, *on average*, associated with higher or lower levels of some outcome. Similar to other studies our analyses focus on the most common genetic variants—so called single-nucleotide polymorphisms (SNPs). SNPs are sites in the genome where single DNA base pairs commonly differ across individuals. SNPs usually have two different possible base pairs, or alleles. Although there are tens of millions of sites where SNPs are located in the human genome, GWASs typically investigate only SNPs that can be measured (or imputed) with a high level of accuracy. Currently, such procedures usually yield millions of SNPs that together capture the most common genetic variations across people.

GWAS has been a successful research strategy for identifying genetic variants associated with many traits and diseases, including body height[58–60], Alzheimer's disease[61,62], and schizophrenia[63,64]. It has also recently been used to identify genetic variants associated with a variety of health-relevant social science outcomes, such as the number of children a person has[65], happiness[66,67], and educational attainment[31,66,68].

Statistical geneticists would call a genetic variant causal if a *ceteris paribus* change in that variant at conception would lead to a different outcome (e.g., higher income). Note that this definition of causality requires no minimum effect size (i.e., the change in income due to the change in the genetic variant may so small that it is barely measurable), nor does it require an understanding of the mechanism (i.e., the genetic effect may be indirect and mediated by environmental responses, self-selection into environments and so forth).

GWAS identifies genetic variants that are associated with the outcome, but an observed association with a specific variant does not imply that the variant *causes* the outcome, for a variety of reasons. First, genetic variants are often highly correlated with other, nearby variants on the same chromosome. As a result, when one or more variants in a region causally influence an outcome (in that particular environment), many noncausal variants in that region may also be identified as associated with the outcome. When GWAS results are analyzed, researchers often emphasize results for the genetic variant in a region that showed the strongest evidence of association. This variant does not need to be the causal variant. In fact, the causal genetic variant may not have even been measured directly. For example, GWAS that focus on common SNPs would not be able to identify rare or structural genetic

variants (e.g., deletions or insertions of an entire genetic region) that are causal, but they may identify SNPs that are correlated with these unobserved variants.

Second, the frequencies of many genetic variants vary systematically across environments. If those environmental factors are not accounted for in the association analyses, some of the associations found may be spurious. To use a well-known example,[69] any genetic variants common in people of Asian ancestries will be associated statistically with chopstick use, but these variants would not *cause* chopstick use; rather, these genetic variants and the outcome of chopstick use are both distributed unevenly among people with different ancestries. This is the problem of "population stratification". GWAS researchers have a number of strategies for addressing the challenges posed by population stratification (see the FAQ section Quality control measures).

Even in studies such as ours that attempt to address and correct for heterogeneity in genetic ancestry, allele frequencies may nonetheless vary systematically with environmental factors. For example, a genetic variant that is associated with higher income in the parental generation may have downstream effects on children's educational outcomes (e.g., through living in a neighborhood with good schools). This same genetic variant is likely to be inherited by the children of these parents, creating a correlation between the presence of the genetic variant in a child's genome and the extent to which the child was reared in an environment with specific characteristics. A recent study of Icelandic families showed that the parental allele that is *not* passed on to the parent's offspring is still associated with the child's educational attainment, suggesting that GWAS results for educational attainment partly represent these intergenerational pathways.[16] Our sibling analyses yield results that are consistent with this conclusion.

Third, variants' effects on an outcome may be indirect, so a variant that may be "causal" in one environment may have a diminished effect or no effect at all in other environments. For example, the nicotinic acetylcholine receptor gene cluster on chromosome 15 is associated with lung cancer.[70–72] From this observation alone we cannot conclude that these genetic variants cause lung cancer through some direct biological mechanism. In fact, it is likely that these genetic variants increase lung cancer risk through their effects on smoking behavior. In a tobacco-free environment, it is plausible that many of these associations would be substantially weaker and perhaps disappear altogether. Thus, even *if* we have credible evidence that a specific association is not spurious, it is entirely possible that the genetic variant in question influences the outcome through channels that we, in common parlance, would label environmental (e.g., smoking). Nearly forty years ago, the sociologist Christopher Jencks criticized the widespread tendency to mistakenly treat environmental and genetic sources of variation as mutually exclusive, see also [57]. As the example of smoking illustrates, it is often overly simplistic to assume that "genetic explanations of

behavior are likely to be exclusively physical explanations while environmental explanations are likely to be social".[28]

5.3. What is a polygenic index?

The results of GWAS can be used to create a "polygenic index" (often also called a polygenic score), an index composed of many genetic variants from across the genome. More precisely, GWAS results are used to create a *formula* for how to construct a polygenic index. Using this formula, a polygenic index can then be constructed for any individual with genome-wide data. Indeed, some of the value of GWAS is that the polygenic index it produces can be used in subsequent studies conducted in other samples.

Because a polygenic index aggregates the information from many genetic variants, it is more strongly associated with variation among individuals for the GWAS outcome than with any single genetic variant. Often, the polygenic indices that are most strongly associated with an outcome are those created using *all* common genetic variants (typically more than a million) studied in GWAS. The larger the GWAS sample is, the more precise the polygenic index constructed from the GWAS results will be. If GWAS samples were infinitely large, the polygenic indices constructed from those samples would be expected to capture as much of the variance of a trait in an independent sample as implied by the SNP heritability of a trait. For example, in our study, we estimated the SNP heritability for income to be approximately 10%. That means that a polygenic index constructed from an infinitely large GWAS on income would be expected to capture approximately 10% of the variance in income in an independent sample that was not included in the GWAS.[73,74]

Note that this does not mean that 10% of anyone's income is biologically determined (see the FAQ sections What does your study not mean? and Does this study show that an individual's level of income, education, or health is determined, or fixed, at conception?). It is important to understand that polygenic indices are not a "clean" way to separate biological from non-biological factors that contribute to differences between people's outcomes.[18] GWAS results are not entirely immune to unobserved (e.g., environmental) confounds, such as parenting, or neighborhood characteristics, and genetic influences are often conditional on and/or mediated by environmental channels (What is a GWAS? Are the genetic variants identified in a GWAS "causal"? and [75]). For example, a society that systematically discriminates against people of color would induce a correlation between all genes for skin or hair pigmentation and income. However, a change in that society that would eliminate such discrimination could make these genetic associations disappear. Since polygenic indices merely aggregate the effects that were estimated in a GWAS, they partly reflect currently existing social realities. Furthermore, polygenic indices may exhibit

different predictive accuracy even among members of the same ancestry group that vary from each other in terms of sex or socioeconomic status.[76]

5.4. Where can I learn more about social science genetics?

Two of the coauthors of the current study, Harden and Koellinger, published a review on social science genetics in *Nature Human Behavior* [18] that provides a comprehensive answer. In short, we believe that the social sciences are incomplete without genetics because most differences between people in terms of their behavior, preferences, achievements, and life events are at least to some extent "heritable" (see the FAQ section What does it mean to say that income is "heritable"?). Thus, integrating molecular genetics into the social sciences presents an opportunity to deliver richer, more precise answers to old questions in psychology, sociology, economics and related fields. Furthermore, the new type of data and study designs that molecular genetics allow will grant social scientists opportunities to ask new questions and to pursue new answers that would not have previously been feasible. We see our current study as an example for both of these claims (What did you do in this paper?).

The review by Harden and Koellinger also discusses the challenges of this work more broadly and provides several substantive examples of new insights afforded by integrating molecular genetic data into the social sciences (e.g., with respect to the intergenerational transmission of human capital, social mobility over the lifespan, human demography, gene-environment interactions, and the intersection between "social/behavioral" genetics and "disease" genetics).

6. Appendices

6.1. Quality control measures

There are many potential pitfalls that could lead to spurious results in genome-wide association studies (GWAS). We took many precautions to guard against these pitfalls.

One potential source of spurious results is incomplete "quality control (QC)" of the genetic data. To avoid this problem, we used state-of-the-art QC protocols from medical genetics research.[77] We supplemented these protocols by developing and applying additional, more stringent QC filters.

Another potential source of spurious results is a confound known as "population stratification." To give a well-known illustration, suppose we were conducting a GWAS on the use of chopsticks.[69] People of Asian ancestries are more likely to use chopsticks than people of European ancestries. If we combined samples of Chinese and European ancestries and performed a GWAS that ignores ancestry, then we would find genetic associations for these variants. However, those associations would simply reflect the fact that allele frequencies vary across ancestry groups.

In our study we were extremely careful to correct for population stratification as much as possible. At the outset, we restricted the study to individuals of European ancestries. As is standard in GWAS, we also controlled for "principal components" of the genetic data in the analysis; these principal components capture the small genetic differences across ancestry groups within European populations, so controlling for them largely removes the spurious associations arising solely from these small ancestry differences.

After taking these steps to minimize bias stemming from population stratification, we conducted follow up analysis with ~15,000 pairs of siblings (~30,000 individuals). These "within-family" analyses break the link between genes and the family environment, thanks to the natural experiment of meiosis. During meiosis, the two copies of each parental chromosome are randomly combined and then separated to create a set of two gametes (e.g., two eggs or two sperm) each of which contains only one new, resampled copy of each chromosome. This process creates an almost infinite number of different DNA sequences that each parent could theoretically pass on to their children. The resulting genetic differences between full siblings and dizygotic twins are therefore random and independent from environmental factors that vary between families. Therefore, comparisons among biological siblings yield estimates for our polygenic index that are immune to genetic nurture, uncontrolled population structure, and other sorts of environmental influences that cannot be traced back to direct genetic effects. In these analyses, we found that approximately 75% of the signal of our polygenic index is due to such environmental

confounds, while the remaining 25% is due to genetic effects that originate in people we studied. We call these latter effects causal, even though the pathways from genes to outcomes are complex and often work via environmental channels (e.g. education, interactions with peers).

6.2. Additional reading and references

Note: Whenever possible, we included links to freely available versions of these references.

1. Demange, P. A. et al. Investigating the genetic architecture of non-cognitive skills using GWAS-by-subtraction. bioRxiv 2020.01.14.905794 (2020) doi:10.1101/2020.01.14.905794.
2. Piketty, T. Social Mobility and Redistributive Politics. Q. J. Econ. 110, 551–584 (1995).
3. Wilkinson, R. G. & Marmot, M. Social Determinants of Health: The Solid Facts. (World Health Organization, 2003).
4. Stringhini, S. et al. Socioeconomic status and the 25× 25 risk factors as determinants of premature mortality: a multicohort study and meta-analysis of 1\textperiodcentered 7 million men and women. Lancet 389, 1229–1237 (2017).
5. Stevenson, B. & Wolfers, J. Subjective Well-Being and Income: Is There Any Evidence of Satiation? Am. Econ. Rev. 103, 598–604 (2013).
6. Adler, N. E. et al. Socioeconomic status and health. The challenge of the gradient. American Psychology 49, 15–24 (1994).
7. Heckman, J. J. & Mosso, S. The Economics of Human Development and Social Mobility. Annu. Rev. Econom. 6, 689–733 (2014).
8. Haveman, R. & Smeeding, T. The role of higher education in social mobility. Future Child. 16, 125–150 (2006).
9. Acemoglu, D. Technical Change, Inequality, and the Labor Market. J. Econ. Lit. 40, 7–72 (2002).
10. Plomin, R., DeFries, J. C., Knopik, V. S. & Neiderhiser, J. M. Behavioral Genetics. (Worth Publishers, 2012).
11. Okbay, A. et al. Polygenic prediction of educational attainment within and between families from genome-wide association analyses in 3 million individuals. Nat. Genet. 54, 437–449 (2022).
12. Polderman, T. J. C. et al. Meta-analysis of the heritability of human traits based on fifty years of twin studies. Nat. Genet. 47, 702–709 (2015).
13. Krapohl, E. & Plomin, R. Genetic link between family socioeconomic status and children's educational achievement estimated from genome-wide SNPs. Mol. Psychiatry 21, 437–443 (2016).
14. Horn, J. M., Loehlin, J. C. & Willerman, L. Intellectual resemblance among adoptive adoptive and biological relatives: the Texas adoption project. Behav. Genet. 9, 177–201 (1979).
15. D'Onofrio, B. M. et al. The role of the children of twins design in elucidating causal relations between parent characteristics and child outcomes. J. Child Psychol.

Psychiatry 44, 1130–1144 (2003).
16. Kong, A. et al. The nature of nurture: Effects of parental genotypes. Science 359, 424–428 (2018).
17. DiPrete, T. A., Burik, C. A. P. & Koellinger, P. D. Genetic instrumental variable regression: Explaining socioeconomic and health outcomes in nonexperimental data. Proceedings of the National Academy of Sciences of the United States of America 115, E4970–E4979 (2018).
18. Harden, K. P. & Koellinger, P. D. Using genetics for social science. Nature Human Behaviour 4, 567–576 (2020).
19. Visscher, P. M. et al. 10 Years of GWAS Discovery: Biology, Function, and Translation. Am. J. Hum. Genet. 101, 5–22 (2017).
20. Chabris, C. F., Lee, J. J., Cesarini, D., Benjamin, D. J. & Laibson, D. I. The fourth law of behavior genetics. Curr. Dir. Psychol. Sci. 24, 304–312 (2015).
21. Dougherty, C. Why are the returns to schooling higher for women than for men? J. Hum. Resour. XL, 969–988 (2005).
22. Kevles, D. J. In the Name of Eugenics: Genetics and the Uses of Human Heredity. (Harvard University Press, 1995).
23. Moore, T. O. Stamped from the Beginning: The Definitive History of Racist Ideas in America, by Ibram X. Kendi. Black Scholar 48, 71–74 (2018).
24. Crouch, C. Molly Ladd-Taylor: Fixing the Poor: Eugenic Sterilization and Child Welfare in the Twentieth Century. Preprint at https://idp.springer.com/authorize/casa?redirect_uri=https://link.springer.com/article/10.1007/s10964-020-01218-w&casa_token=vQPCN-yroYoAAAAA:iuJEb2oPmHxKeorxPy59lLCGUtrAXqo1kmyB9LYUgXDhht4O3163bqZieggTcM1Ec_R-TVKRk5X8U2w (2020).
25. Carlson, E. A. Three Generations, No Imbeciles: Eugenics, the Supreme Court, and Buck v. Bell. By Paul A. Lombardo. Baltimore (Maryland): Johns Hopkins University Press. $29.95. xv 365 p.; ill.; index. 978-0-8018-9010-9. 2008. The Quarterly Review of Biology vol. 84 178–180 Preprint at https://doi.org/10.1086/603452 (2009).
26. Hill, W. D. et al. Molecular genetic contributions to social deprivation and household income in UK Biobank. Curr. Biol. 26, 3083–3089 (2016).
27. Hill, W. D. et al. Genome-wide analysis identifies molecular systems and 149 genetic loci associated with income. Nat. Commun. 10, 5741 (2019).
28. Jencks, C. Heredity, environment, and public policy reconsidered. Am. Sociol. Rev. 45, 723–736 (1980).
29. Goldberger, A. S. Heritability. Economica 46, 327–347 (1979).
30. Martin, A. R. et al. Clinical use of current polygenic risk scores may exacerbate health disparities. Nat. Genet. 51, 584–591 (2019).
31. Lee, J. J. et al. Gene discovery and polygenic prediction from a genome-wide association study of educational attainment in 1.1 million individuals. Nat. Genet. 50, 1112–1121 (2018).
32. 1000 Genomes Project Consortium et al. A global reference for human genetic variation. Nature 526, 68–74 (2015).
33. Domingue, B. W., Belsky, D., Conley, D., Harris, K. M. & Boardman, J. D. Polygenic Influence on Educational Attainment: New evidence from The National Longitudinal Study of Adolescent to Adult Health. AERA Open 1, 1–13 (2015).
34. Vassos, E. et al. An Examination of Polygenic Score Risk Prediction in Individuals With First-Episode Psychosis. Biol. Psychiatry 81, 470–477 (2017).
35. Domingue, B. W. et al. Mortality selection in a genetic sample and implications for association studies. International Journal of Epidemiology vol. 46 1285–1294 Preprint

at https://doi.org/10.1093/ije/dyx041 (2017).
36. Belsky, D. W. et al. Development and evaluation of a genetic risk score for obesity. Biodemography Soc. Biol. 59, 85–100 (2013).
37. Mills, M. C. & Rahal, C. A scientometric review of genome-wide association studies. Commun. Bio. 2, 9 (2019).
38. Harden, K. P. The Genetic Lottery: Why DNA Matters for Social Equality. (Princeton University Press, 2021).
39. Rawls, J. A theory of justice. (Belknap Press of Harvard University Press, 1999).
40. Roemer, J. E. Equality of Opportunity. (Harvard University Press, 1998).
41. Gromet, D. M., Hartson, K. A. & Sherman, D. K. The politics of luck: Political ideology and the perceived relationship between luck and success. J. Exp. Soc. Psychol. 59, 40–46 (2015).
42. Cappelen, A. W., Konow, J., Sørensen, E. Ø. & Tungodden, B. Just luck: An experimental study of risk-taking and fairness. Am. Econ. Rev. 103, 1398–1413 (2013).
43. Almås, I., Cappelen, A. W., Sørensen, E. Ø. & Tungodden, B. Fairness and the development of inequality acceptance. Science 328, 1176–1178 (2010).
44. Cappelen, A. W., Sørensen, E. Ø. & Tungodden, B. Responsibility for what? Fairness and individual responsibility. Eur. Econ. Rev. 54, 429–441 (2010).
45. Alesina, A. & Ferrara, E. L. Preferences for redistribution in the land of opportunities. Journal of Public Economics 89, 897–931 (2005).
46. Alesina, A., Stantcheva, S. & Teso, E. Intergenerational mobility and preferences for redistribution. Am. Econ. Rev. 108, 521–554 (2018).
47. Nozick, R. Anarchy, state, and utopia. vol. 5038 (New York: Basic Books, 1974).
48. Joly, Y. et al. Establishing the International Genetic Discrimination Observatory. Nat. Genet. 52, 466–468 (2020).
49. Benjamin, D. J. et al. The genetic architecture of economic and political preferences. PNAS 109, 8026–8031 (2012).
50. van der Loos, M. J. H. M. et al. Candidate gene studies and the quest for the entrepreneurial gene. Small Bus. Econ. 37, 269–275 (2011).
51. Beauchamp, J. P. et al. Molecular Genetics and Economics. J. Econ. Perspect. 25, 57–82 (2011).
52. Karlsson Linnér, R. et al. Genome-wide association analyses of risk tolerance and risky behaviors in over 1 million individuals identify hundreds of loci and shared genetic influences. Nat. Genet. 51, 245–257 (2019).
53. Hewitt, J. K. Editorial policy on candidate gene association and candidate gene-by-environment interaction studies of complex traits. Behav. Genet. 42, 1–2 (2012).
54. Bioethics, N. C. on. Genetics and Human Behavior: The Ethical Context. (Nuffield Council on Bioethics, 2002).
55. Visscher, P. M., Hill, W. G. & Wray, N. R. Heritability in the genomics era--concepts and misconceptions. Nat. Rev. Genet. 9, 255–266 (2008).
56. Taubman, P. The determinants of earnings: Genetics, family, and other environments: A study of white male twins. Am. Econ. Rev. 66, 858–870 (1976).
57. Turkheimer, E. Three laws of behavior genetics and what they mean. Curr. Dir. Psychol. Sci. 9, 160–164 (2000).
58. Wood, A. R. et al. Defining the role of common variation in the genomic and biological architecture of adult human height. Nat. Genet. 46, 1173–1186 (2014).
59. Locke, A. E. et al. Genetic studies of body mass index yield new insights for obesity biology. Nature 518, 197–206 (2015).
60. Yengo, L. et al. Meta-analysis of genome-wide association studies for height and

body mass index in ~700,000 individuals of European ancestry. Human Molecular Genetics 27, 3641–3649 (2018).
61. Jansen, I. E. et al. Genetic meta-analysis identifies 9 novel loci and functional pathways for Alzheimer's disease risk. bioRxiv 258533 (2018) doi:10.1101/258533.
62. Lambert, J. C. et al. Meta-analysis of 74,046 individuals identifies 11 new susceptibility loci for Alzheimer's disease. Nat. Genet. 45, 1452–1458 (2013).
63. Ripke, S. et al. Biological insights from 108 schizophrenia-associated genetic loci. Nature 511, 421–427 (2014).
64. Pardiñas, A. F. et al. Common schizophrenia alleles are enriched in mutation-intolerant genes and in regions under strong background selection. Nat. Genet. 50, 381–389 (2018).
65. Barban, N. et al. Genome-wide analysis identifies 12 loci influencing human reproductive behavior. Nat. Genet. 48, 1462–1472 (2016).
66. Okbay, A. et al. Genetic variants associated with subjective well-being, depressive symptoms, and neuroticism identified through genome-wide analyses. Nat. Genet. 48, 624–633 (2016).
67. Turley, P. et al. Multi-trait analysis of genome-wide association summary statistics using MTAG. Nat. Genet. 50, 229–237 (2018).
68. Rietveld, C. A. et al. GWAS of 126,559 individuals identifies genetic variants associated with educational attainment. Science 340, 1467–1471 (2013).
69. Lander, E. S. & Schork, N. J. Genetic dissection of complex traits. Science 265, 2037–2048 (1994).
70. Thorgeirsson, T. E. et al. A variant associated with nicotine dependence, lung cancer and peripheral arterial disease. Nature 452, 638–642 (2008).
71. Amos, C. I. et al. Genome-wide association scan of tag SNPs identifies a susceptibility locus for lung cancer at 15q25.1. Nat. Genet. 40, 616–622 (2008).
72. Hung, R. J. et al. A susceptibility locus for lung cancer maps to nicotinic acetylcholine receptor subunit genes on 15q25. Nature 452, 633–637 (2008).
73. Daetwyler, H. D., Villanueva, B. & Woolliams, J. A. Accuracy of predicting the genetic risk of disease using a genome-wide approach. PLoS One 3, e3395 (2008).
74. de Vlaming, R. et al. Meta-GWAS accuracy and power (MetaGAP) calculator shows that hiding heritability is partially due to imperfect genetic correlations across studies. PLoS Genet. 13, (2017).
75. Young, A. I., Benonisdottir, S., Przeworski, M. & Kong, A. Deconstructing the sources of genotype-phenotype associations in humans. Science 365, 1396–1400 (2019).
76. Mostafavi, H. et al. Variable prediction accuracy of polygenic scores within an ancestry group. Elife 9, (2020).
77. Winkler, T. W. et al. Quality control and conduct of genome-wide association meta-analyses. Nat. Protoc. 9, 1192–1212 (2014).

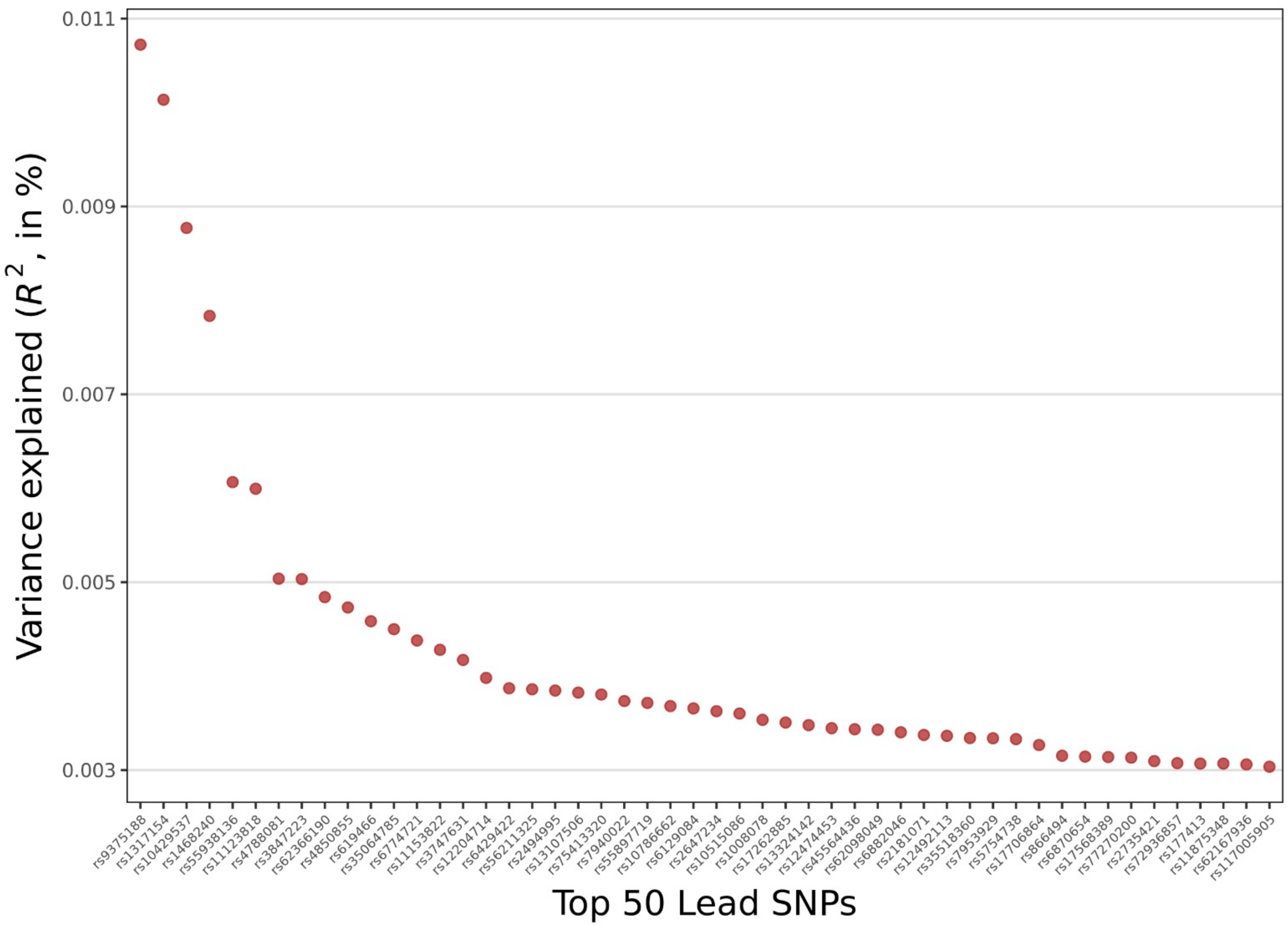

Supplementary Fig. 1. Effect sizes of Income Factor GWAS

Each point represents the effect size (R^2) for the top 50 lead SNPs from the Income Factor GWAS, adjusted for winner's curse.

1. Individual income

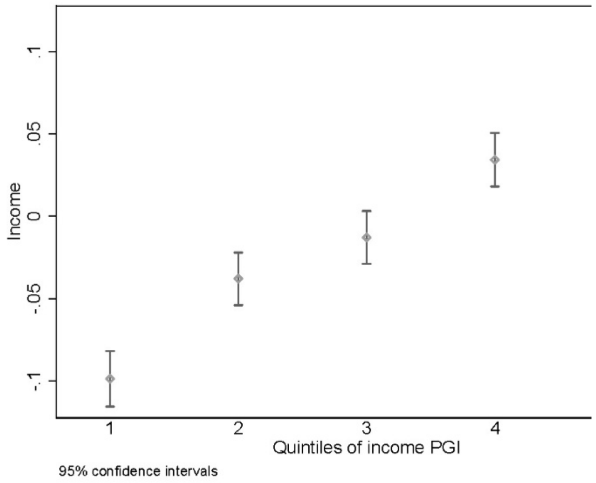

2. Occupational income

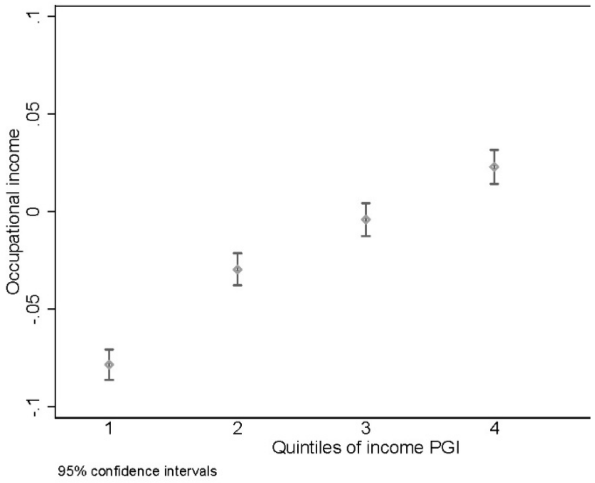

3. Household income

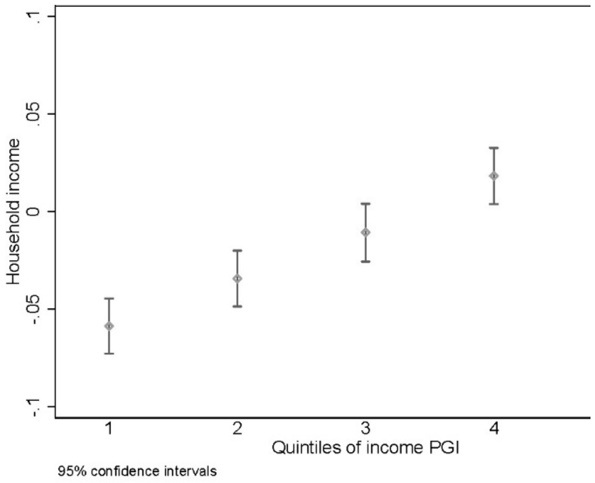

Supplementary Fig 2. Polygenic prediction of income

Panel (1) - (3) show average levels of individual/occupational/household income per PGI quintile in STR, along with 95% confidence intervals. The analyses contain *N* = 28,359 / 21,990 / 17,418 observations, respectively. Outcomes were first residualised on sex and the first 20 principal components and then normalised to have a mean zero and standard deviation of one.

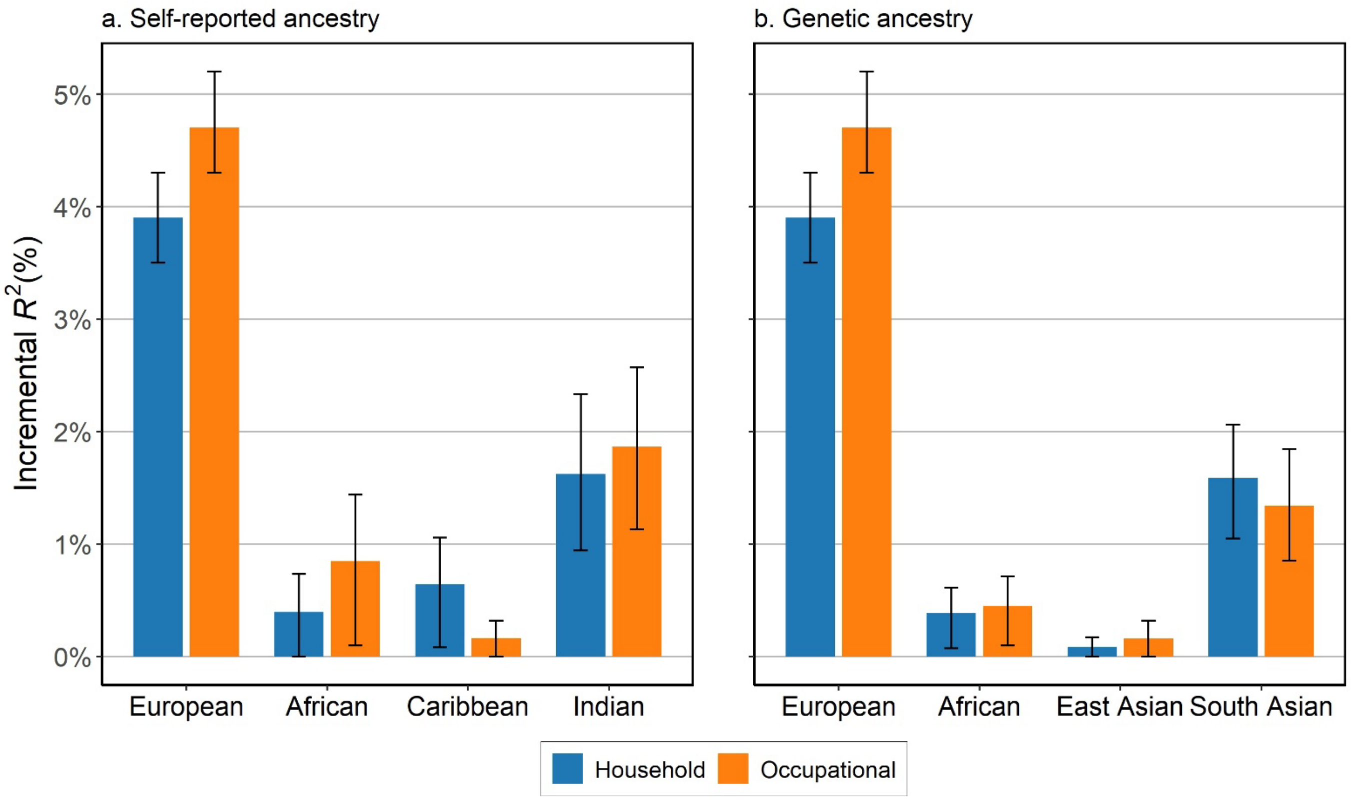

Supplementary Fig. 3. Polygenic prediction of income in samples of non-European ancestries

The figures report results in the UK Biobank (UKB), comparing the predictive accuracy of the Income Factor PGI in samples of European ancestry with those of non-European ancestry. The left panel defines ancestry groups based on self-reports (UKB data field 21000), showing results for the three largest non-EUR groups ($N_{African}$ = 3,210, $N_{Caribbean}$ = 4,300, and N_{Indian} = 5,661). The right panel defines the non-European ancestry groups based on observed genetic ancestries ($N_{Africans}$ = 9,494, $N_{East\text{-}Asians}$ = 2,216, and $N_{South\text{-}Asian}$ = 11,413). Before fitting the regressions, each phenotype was residualised of demographic covariates (sex, a third-degree polynomial in age, and interactions with sex). Incremental R^2 is the difference between the R^2 from regressing the residualised outcome on the PGI and the controls (20 genetic PCs and genotyping batch indicators) and the R^2 from a regression only on the controls. The error bars indicate 95% confidence intervals obtained by bootstrapping the sample 1,000 times.
