# Supplementary material for: Associations between common genetic variants and income provide insights about the socio-economic health gradient
Source: Nat Hum Behav. 2025 Jan 28;9(4):794–805. doi: 10.1038/s41562-024-02080-7 (PMC12018258; doi:10.1038/s41562-024-02080-7)
Supplement: Supplementary file 2 — Reporting Summary [file 41562_2024_2080_MOESM2_ESM.pdf]

nature portfolio

Corresponding author(s): Philipp Koellinger

Last updated by author(s): Oct 20, 2024

Reporting Summary

Nature Portfolio wishes to improve the reproducibility of the work that we publish. This form provides structure for consistency and transparency in reporting. For further information on Nature Portfolio policies, see our Editorial Policies and the Editorial Policy Checklist.

Statistics

For all statistical analyses, confirm that the following items are present in the figure legend, table legend, main text, or Methods section.

n/a	Confirmed	
☐	☒	The exact sample size (*n*) for each experimental group/condition, given as a discrete number and unit of measurement
☐	☒	A statement on whether measurements were taken from distinct samples or whether the same sample was measured repeatedly
☐	☒	The statistical test(s) used AND whether they are one- or two-sided<br>*Only common tests should be described solely by name; describe more complex techniques in the Methods section.*
☐	☒	A description of all covariates tested
☐	☒	A description of any assumptions or corrections, such as tests of normality and adjustment for multiple comparisons
☐	☒	A full description of the statistical parameters including central tendency (e.g. means) or other basic estimates (e.g. regression coefficient) AND variation (e.g. standard deviation) or associated estimates of uncertainty (e.g. confidence intervals)
☐	☒	For null hypothesis testing, the test statistic (e.g. *F*, *t*, *r*) with confidence intervals, effect sizes, degrees of freedom and *P* value noted<br>*Give P values as exact values whenever suitable.*
☒	☐	For Bayesian analysis, information on the choice of priors and Markov chain Monte Carlo settings
☒	☐	For hierarchical and complex designs, identification of the appropriate level for tests and full reporting of outcomes
☐	☒	Estimates of effect sizes (e.g. Cohen's *d*, Pearson's *r*), indicating how they were calculated

Our web collection on statistics for biologists contains articles on many of the points above.

Software and code

Policy information about availability of computer code

Data collection	N/A (Our study is entirely based on previously existing data)
Data analysis	METAL, release 2011-03-25, http://csg.sph.umich.edu/abecasis/metal/; MTAG software v.1.0.1, https://github.com/omeed-maghzian/mtag; GSEM v.0.0.3e https://github.com/GenomicSEM/GenomicSEM/wiki; LDSC and LDSC-SEG v1.0.1 https://github.com/bulik/ldsc; MAGMA v1.10 https://ctg.thebluebus.nl/software/magma; COJO and GREML in GCTA v1.94 https://yanglab.westlake.edu.cn/software/gcta/#Download; LDpred2 https://github.com/privefl/paper-ldpred2/tree/master; FUMA https://fuma.ctglab.nl/tutorial#overview, EasyQC 23.8 https://www.uni-regensburg.de/medizin/epidemiologie-praeventivmedizin/genetische-epidemiologie/software/index.html, snipar https://github.com/AlexTISYoung/snipar

For manuscripts utilizing custom algorithms or software that are central to the research but not yet described in published literature, software must be made available to editors and reviewers. We strongly encourage code deposition in a community repository (e.g. GitHub). See the Nature Portfolio guidelines for submitting code & software for further information.

Data

Policy information about availability of data

All manuscripts must include a data availability statement. This statement should provide the following information, where applicable:

- Accession codes, unique identifiers, or web links for publicly available datasets
- A description of any restrictions on data availability
- For clinical datasets or third party data, please ensure that the statement adheres to our policy

GWAS summary statistics are available at https://beta.dpid.org/149. Data for our analyses come from many cohorts and organizations, some of which are subject to a MTA, and are listed in the Supplementary Information and Supplementary Table 1. Individual-level data are subject to privacy restrictions and can be requested directly from the participating cohorts.

Research involving human participants, their data, or biological material

Policy information about studies with human participants or human data. See also policy information about sex, gender (identity/presentation), and sexual orientation and race, ethnicity and racism.

Reporting on sex and gender	We conducted sex-stratified GWAS on income in all participating samples. Sex-stratified analyses are clearly described as such in the manuscript and supplementary information. We share both sex-stratified and aggregated GWAS summary statistics publicly.
Reporting on race, ethnicity, or other socially relevant groupings	We restricted our analyses to 1000 Genomes EUR-like individuals to maximize statistical power and to minimize bias from unobserved environmental factors that are correlated with differences in minor allele frequencies across ancestry groups. As with previous genetic studies on social outcomes like educational attainment, the findings of this study have limited generalisability across different populations.
Population characteristics	We restricted our analyses to 1KG-EUR-like individuals who were not currently enrolled in an educational program or who were aged above 30 if their current enrollment status was unknown.
Recruitment	Recruitment protocols varied across participating cohorts and are described in the references provided for each dataset (Supplementary Table 1).
Ethics oversight	Each participating cohort signed a collaboration agreement verifying that the responsible Institutional Review Board (IRB) or ethical committee has approved a GWAS of income in that sample.

Note that full information on the approval of the study protocol must also be provided in the manuscript.

Field-specific reporting

Please select the one below that is the best fit for your research. If you are not sure, read the appropriate sections before making your selection.

☐ Life sciences ☒ Behavioural & social sciences ☐ Ecological, evolutionary & environmental sciences

For a reference copy of the document with all sections, see nature.com/documents/nr-reporting-summary-flat.pdf

Behavioural & social sciences study design

All studies must disclose on these points even when the disclosure is negative.

Study description	We conducted sex-stratified GWAS and meta-analyzed results from 32 cohorts across 12 economically advanced countries and three continents, yielding the largest GWAS on income to date with an effective sample size of N = 668,288 (Table 1).
Research sample	We restricted our analyses to 1000 Genomes EUR-like individuals to maximize statistical power and to minimize bias from unobserved environmental factors that are correlated with differences in minor allele frequencies across ancestry groups. As with previous genetic studies on social outcomes like educational attainment, the findings of this study have limited generalisability across different populations.
Sampling strategy	Sampling strategies varied across participating cohorts and are described in the references provided for each dataset (Supplementary Table 1).
Data collection	Data collection strategies varied across participating cohorts and are described in the references provided for each dataset (Supplementary Table 1).
Timing	Timing of data collection varied across participating cohorts and are described in the references provided for each dataset (Supplementary Table 1).
Data exclusions	We restricted our analyses to 1000 Genomes EUR-like individuals to maximize statistical power and to minimize bias from

Data exclusions	unobserved environmental factors that are correlated with differences in minor allele frequencies across ancestry groups.
Non-participation	N/A (secondary data analyses)
Randomization	N/A

Reporting for specific materials, systems and methods

We require information from authors about some types of materials, experimental systems and methods used in many studies. Here, indicate whether each material, system or method listed is relevant to your study. If you are not sure if a list item applies to your research, read the appropriate section before selecting a response.

Materials & experimental systems

n/a	Involved in the study
☒	☐ Antibodies
☒	☐ Eukaryotic cell lines
☒	☐ Palaeontology and archaeology
☒	☐ Animals and other organisms
☒	☐ Clinical data
☒	☐ Dual use research of concern
☒	☐ Plants

Methods

n/a	Involved in the study
☒	☐ ChIP-seq
☒	☐ Flow cytometry
☒	☐ MRI-based neuroimaging

Plants

Seed stocks	N/A
Novel plant genotypes	N/A
Authentication	N/A
